# The Beta Cell in Its Cluster: Stochastic Graphs of Beta Cell Connectivity in the Islets of Langerhans

**DOI:** 10.1371/journal.pcbi.1004423

**Published:** 2015-08-12

**Authors:** Deborah A. Striegel, Manami Hara, Vipul Periwal

**Affiliations:** 1 Laboratory of Biological Modeling, National Institute of Diabetes and Digestive and Kidney Diseases, National Institutes of Health, Bethesda, Maryland, United States of America; 2 Department of Medicine, University of Chicago, Chicago, Illinois, United States of America; University of Padua, ITALY

## Abstract

Pancreatic islets of Langerhans consist of endocrine cells, primarily α, β and δ cells, which secrete glucagon, insulin, and somatostatin, respectively, to regulate plasma glucose. β cells form irregular locally connected clusters within islets that act in concert to secrete insulin upon glucose stimulation. Due to the central functional significance of this local connectivity in the placement of β cells in an islet, it is important to characterize it quantitatively. However, quantification of the seemingly stochastic cytoarchitecture of β cells in an islet requires mathematical methods that can capture topological connectivity in the entire β-cell population in an islet. Graph theory provides such a framework. Using large-scale imaging data for thousands of islets containing hundreds of thousands of cells in human organ donor pancreata, we show that quantitative graph characteristics differ between control and type 2 diabetic islets. Further insight into the processes that shape and maintain this architecture is obtained by formulating a stochastic theory of β-cell rearrangement in whole islets, just as the normal equilibrium distribution of the Ornstein-Uhlenbeck process can be viewed as the result of the interplay between a random walk and a linear restoring force. Requiring that rearrangements maintain the observed quantitative topological graph characteristics strongly constrained possible processes. Our results suggest that β-cell rearrangement is dependent on its connectivity in order to maintain an optimal cluster size in both normal and T2D islets.

## Author Summary

High or low blood glucose levels are detrimental to human health. The hormone-secreting cells primarily responsible for maintaining glucose at physiologically appropriate levels are embedded in small clusters within the pancreas, the so-called islets of Langerhans. These islets have an irregular arrangement of cells, β cells that secrete insulin, α cells that secrete glucagon, and other cells with less well-understood functions. While the arrangement of β cells is irregular, these cells need to be touching for the islet to respond to glucose with insulin secretion. We first use a mathematical formalism called graph theory to show that cell arrangements in islets from diabetic and control donors are significantly different. The question we then address is: Is there some set of moves of islet cells that will preserve the observed arrangement? The aim is to gain insight into the biological processes by which islets are formed and maintained. We find moves on β-cell graphs that leave the same significant aspects of cell arrangements unchanged. These moves turn out to be severely restricted, and suggest that β cells may prefer to move from larger clusters but can move to a cluster of any size, possibly to maximize their exposure to blood vessels.

## Introduction

Pancreatic islets of Langerhans make up 2% of the average pancreatic mass (or 0.000028% of human body mass), yet contribute significantly to the regulation of blood glucose levels. These micro-organs consist primarily of α, β, and δ cells that produce the hormones glucagon, insulin, and somatostatin, respectively.

β-β cell contacts are necessary for proper islet function [1–3]. Their electrical coupling allows for the synchronization of intercellular [Ca^2+^] oscillations which results in pulsatile insulin release upon glucose stimulation [4–8] and increases insulin production two-fold as compared to isolated β cells [9] (which show partial recovery in insulin production after reaggregation [10]). This coupling is dependent on Connexin36 (Cx36) gap junction channels [11, 12] since Cx36-deficient mice show altered insulin pulse dynamics and glucose intolerance [13]. Prediabetic mice display impaired Cx36 coupling [14] suggesting a possible role in the progression to T2D. In humans, β cells contain Cx36 gap junctions and levels of Cx36 mRNA correlate with insulin expression [15]. However, Cx36 knockdown reduces incretin-stimulated, but not glucose-stimulated, insulin secretion [16] suggesting the importance of Cx36 may be not through glucose response but through the response to incretins which itself is disrupted by lipotoxicity. Interestingly, the upregulation of Cx36 occurs in unison with the main wave of β-cell differentiation [17], further illustrating the possible dependence of β-cell function on gap junction coupling. It has also been shown that Cx36 protects β cells from apoptosis under cell injury [18] in mice.

Human islets have a unique cytoarchitecture with direct consequences on islet function [19]. The islet’s cytoarchitecture creates the anatomical basis for functional coupling between β cells [20]. However, what is the correct architecture for optimal function of pancreatic islet cells? Qualitatively, this arrangement is non-random [21] and species-dependent [19, 22], with rodent islets displaying a β-cell core surrounded by an α-δ mantle and human islets varying in arrangement in a size-dependent manner [21, 23, 24]. Smaller human islets (effective diameter < 60 μm) consist of an inner core of β cells surrounded by an α- and δ-cell mantle (similar to rodents), whereas in larger islets, α and δ cells are also found within the core possibly due to fusion of subunits [25] or lobules [26] consisting of the mantle-core architecture arranged around penetrating blood vessels [26, 27].

Rare cellular replication and apoptotic events balance β-cell mass over a lifespan [28]. However, with sustained hyperglycemia, changes in β-cell mass have been observed. As metabolic load increases, a complementary increase in β-cell mass is detected in mice [29], rats [30], and humans [31–33]. In mice, β-cell mass increase is achieved through β-cell replication [34], yet in humans β-cell replication is not detectably increased [35, 36], leading some to suggest β cells form under a different mechanism [36]. Nestin-positive islet-derived progenitor cells, which can differentiate into insulin-producing cells, are found in rat and human islets [37]. However, Street et al. [38] found that nestin-positive cells in adult human islets were not colocalized with insulin positive cells suggesting nestin is not expressed in the mature human β cell. With T2D, this initial increase is followed by β-cell mass loss [36] and functional failure believed to be from glucolipotoxicity [14]. Inflammation also affects β-cell mass whereby low concentrations of interleukin-1β (IL-1β) promote β-cell function and prolonged high glucose exposure increases IL-1β which, in turn, increases Fas-mediated apoptosis [39].

β-cell migration is also suggested to occur, most notably during islet development but has also been shown in the adult pancreas. Cole et al. [40] demonstrated that individual human β cells, previously suggested by [41], and large aggregates of cells can form within the ductal epithelium and migrate during gestation. They also found Vimentin (a mesenchymal protein) positive adult human β cells suggesting that adult β cells are capable of remodeling. Recently it was shown that loss of Fbw7 induces pancreatic ductal stem cells to proliferate into endocrine cells in the adult human pancreas [42], with evidence for subsequent migration into islets. Note that the coordinated activation of genes essential for endocrine cell proliferation, migration, vasculogenesis and hormone secretion has been demonstrated [43]. Thus, overall, the processes that govern human β-cell mass maintenance are not well understood. Supporting evidence for any specific process gleaned from rodent models must be considered in the context of possible structural differences between rodent and human islets.

The governing principle determining the optimal arrangement of cells in the healthy and T2D individual is a mystery. Further, there are observed architectural differences in T2D that may contribute to its progression [44], such as β-cell mass loss preferentially in large islets [21], hypertrophy in β cells [45], and amyloid plaque formation in islets [46, 47]. How these architectural changes affect the organization of β cells and their function remains unknown.

Given observations of the results of stochastic development and maintenance processes, there are two basic ways in which one can analyze these processes quantitatively. To set the stage for our islet β-cell analysis, let us consider a hypothetical dataset consisting of a sample of real numbers drawn from a one-dimensional normal distribution. The first standard approach is to make a histogram of the sample, and this histogram recapitulates an approximate normal distribution. A second approach is to consider the numbers as resulting from the equilibrium distribution of a stochastic Langevin process. In this approach, the same sample of normally-distributed random numbers leads us to the Ornstein-Uhlenbeck process with a random walk balancing a linear restoring force in the Langevin dynamics of each number, interpreted as the position of a particle on a line. These two approaches are equally valid views of the data, a static view and a stochastic view, and we shall apply them in turn to the study of β-cell arrangements in islets.

**Fig 1:**
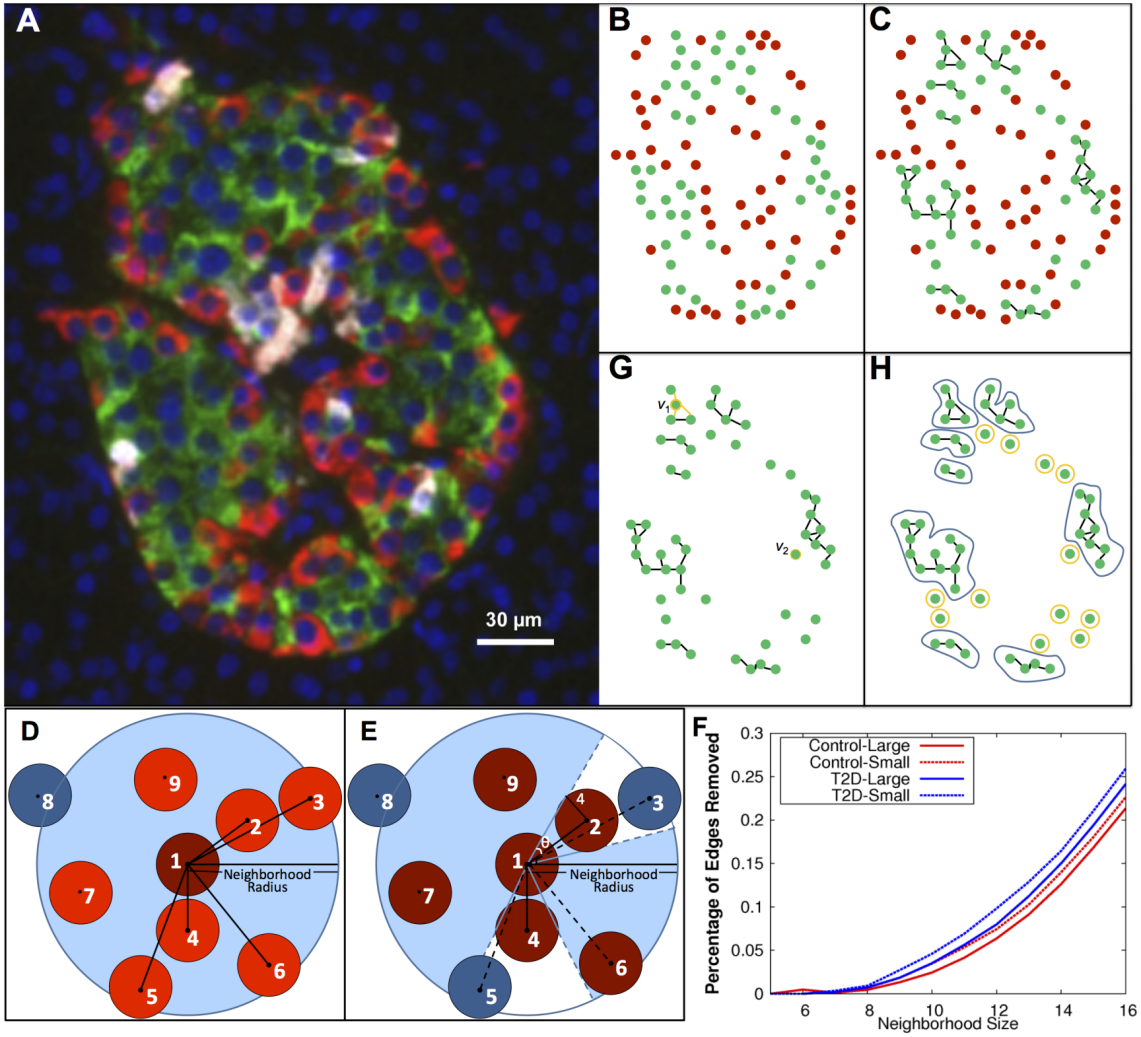
β-cell graphs were created from cells found in human organ donor pancreata and analyzed. The vertices of the graph represent the (*x*,*y*) coordinates of the cells (A,B). For β-cell graphs, vertices were β cells and edges were added between cells within a given neighborhood radius (C). D,E. The neighbors of cells are determined using the shadow algorithm as described in the text, resulting in an increasing number of edges removed as the neighborhood radius increases (F). G. Examples of the degree of cells where *d*(*v*_1_) = 3, *d*(*v*_2_) = 1. H. Example of the components of an islet. Nonsingular and singular components are illustrated with blue and yellow loops, respectively.

We propose that graph theory is an appropriate framework for quantifying β-cell arrangement because it captures quantitative aspects of connectivity flexibly. Graph theory has been used in many contexts in biology, in obvious contexts such as metabolic or regulatory networks, and in more esoteric contexts such as functional MRI analysis [48] and tongue carcinoma prognostication [49]. Geometric graphs have been used to study protein-protein interaction networks [50]. With respect to islets, functional graphs representing β-cell activity in individual mouse islets were created to analyze their small-world characteristics [10]. Images of islet sections show regularities in structure on casual inspection, but the significance of such regularities is difficult to ascertain without a mathematically sound framework for quantifying islet architecture. Here, we apply graph theory to the architecture of islets, and let the vertices of a graph consist of β cells in each islet while the graph's edges represent intercellular connectivity (Fig 1A–1C). This geometric framework captures key architectural characteristics quantitatively in terms of graph-theoretic constructs, as we shall show.

A precondition for applying either approach is the availability of a large-enough dataset. We use the dataset published in [21], consisting of ~9,000 control and ~8,000 T2D two-dimensional sections of islets containing ~200,000 control and ~110,000 T2D cells. All graphs that we consider refer to this two-dimensional dataset, and we are cognizant of the limitations of working with two-dimensional sections of actual three-dimensional structures, the islets. We do address the differences between two-dimensional and three-dimensional graphs of islet cell arrangements by explicitly computing graphs in a smaller islet dataset that does have three-dimensional coordinates for every cell, showing that quantitative measures of these graphs are qualitatively similar. We show first, in the static view, that graph theory quantitative measures, such as mean connectivity of β cells, size of β-cell clusters and number of clusters per islet, can distinguish between control and T2D islets. We then test, in the stochastic view, various models of β-cell degree- and β-cell cluster size-dependent stochastic models of hypothetical graph rearrangements to find possible topological constraints on β-cell rearrangement processes. It is these theoretical relocation processes that are the analogs of the linear restoring force and the random walk defining the normal distribution in the Ornstein-Uhlenbeck example above. To avoid confusion, we emphasize that in the absence of clear experimental evidence on the actual processes that maintain human β cells, our use of verbs such as `move’ is only a theoretical construction. The aim here is to use imaging data to pinpoint the precise mathematical equilibrium processes so that these mathematical constructs can be framed in terms of their contribution to the actual biological realizations, when the experimental data becomes unambiguous.

## Materials and Methods

### Human pancreatic specimens

Twelve T2D and 14 non-diabetic (control) human organ donor specimens were collected and prepared as described in [21]. Briefly, a virtual slice was taken across each pancreatic specimen containing the head, body, and tail regions. This slice was then stained for insulin, glucagon, somatostatin, and DAPI. Tissue sections were imaged and two-dimensional (*x*,*y*) coordinates for each endocrine cell nucleus were recorded using a nucleus marker DAPI with identification of each cell type. The T2D group consisted of 4 males and 8 females ranging in age from 38–81. These subjects died of causes other than diabetes and it is assumed they were normoglycemic with ongoing treatment. The control group had 9 males and 5 females with ages ranging from 15–81. Further information about this dataset can be found in [21]. This dataset, after applying the described imaging method, produced location and cell-type information for ~200,000 cells found in ~9,000 control islets and ~110,000 cells found in ~8,000 T2D islets (Table 1) which was used for further analysis. The control and T2D datasets can be found in S1 Dataset.

**Table 1:** Number of islets and cells per human cadaveric subject. For each control (C) and T2D subject the number of islets (number of large and small islets given in parentheses) and number of cells (number of cells located in large and small islets given in parentheses) are given.

Subj	#Islets (Large, Small)	#Cells (Large, Small)	Subj	#Islets (Large, Small)	#Cells (Large, Small)
C1	332 (81, 251)	5442 (4151, 1291)	T2D1	1059 (157, 902)	11686 (7426, 4260)
C2	705 (246, 459)	16103 (14178, 1925)	T2D2	454 (66, 388)	4645 (2836, 1809)
C3	1372 (417, 955)	26100 (22063, 4037)	T2D3	756 (159, 597)	9109 (6141, 2968)
C4	468 (136, 332)	5864 (4586, 1278)	T2D4	287 (103, 184)	2546 (2032, 514)
C5	200 (110, 90)	14768 (14195, 573)	T2D5	760 (247, 513)	7401 (5526, 1875)
C6	787 (247, 540)	11633 (9581, 2052)	T2D6	216 (60, 156)	3162 (2581, 581)
C7	931 (236, 695)	18985 (15515, 3470)	T2D7	503 (187, 316)	13579 (12017, 1562)
C8	563 (113, 450)	10785 (8842, 1943)	T2D8	459 (155, 304)	8055 (6531, 1524)
C9	755 (236, 519)	16774 (14128, 2646)	T2D9	1448 (276, 1172)	18742 (13902, 4840)
C10	729 (246, 483)	16267 (13478, 2789)	T2D10	393 (105, 288)	10113 (8146, 1967)
C11	502 (233, 269)	18967 (17506, 1461)	T2D11	695 (146, 549)	10355 (6399, 3956)
C12	479 (242, 237)	12369 (11473, 896)	T2D12	899 (225, 674)	11314 (8480, 2834)
C13	864 (279, 585)	16075 (13307, 2768)			
C14	230 (73, 157)	1833 (1412, 421)			
**Total**	**8917 (2895, 6022)**	**191965 (164415, 27550)**	**Total**	**7929 (1886, 6043)**	**110707 (82017, 28690)**

### Graph assignment

Here our focus is on β-cell clusters so henceforth we consider only β cells as the vertices of the graph (Fig 1C). Edges between β cells, as determined by the nearest-neighbors of cells, describe the connectivity of the β-cell cluster.

A cell has a non-zero radius. If we fix a neighborhood size (discussed in the next subsection), for a given cell, not every cell with a center in a neighborhood around this cell is a nearest-neighbor of the cell as not all nearby cells share a contact. The assignment of graphs to the position dataset must take this into account, so that only edges representing nearest-neighbor cell-cell contacts are added to the graph. A typical case is shown in Fig 1D. To determine the neighbors of cell 1, we utilized a shadow algorithm, depicted in Fig 1E, as follows: Neighboring cells were first sorted by distance, then as the sorted list of cells was traversed, edges were added to the graph only if the cell was not in a previous cell’s ‘shadow’. The previous cell’s shadow angle, θ, was calculated by $\theta ={\text{tan}}^{-1}\left(\frac{4}{d}\right)$, where *d* is the distance to the previously added cell and the radius of a cell is 4 μm. In the example in Fig 1D, cell 8 is not a neighbor since the distance between it and cell 1 is greater than the neighborhood radius. After sorting the remaining cells by their distance to cell 1, it is determined that cells 3 and 5 are in the shadow of cells 2 and 4, respectively. Therefore the neighbors of cell 1 are cells 2, 4, 6, 7, and 9 (Fig 1E). Between 2 and 6 percent of total edges are removed from a β-cell graph of neighborhood radius of 10 microns by the shadow algorithm. This percentage increases with respect to an increase in neighborhood radius (Fig 1F). Graphs representing each control and T2D islet were computed. Differences between large and small islets, where small islets are defined by an effective diameter of < 60 microns, have been reported [23]. We will, therefore, analyze the large and small islet groups (defined by the 60 micron diameter) separately as well.

### Neighborhood size from pair distribution functions

While graphs can be deduced with the shadow algorithm for any choice of neighborhood size, it is critical to use the data to determine a range of possible neighborhood sizes. To this end we calculated the pair distribution function, $g\left(r\right)dr=\frac{IsletArea}{2\pi rN^{2}}\sum_{i}\sum_{j\neq i}\delta \left(r-r_{ij}\right)$ where *N* represents the number of vertices, *r*_*ij*_ is the distance between *v*_*i*_ and *v*_*j*_, and $\delta \left(r-r_{ij}\right)=\{\begin{array}{c} 1\;\text{if}\;r=r_{ij}\\ 0\;\text{otherwise} \end{array}$. This function describes how the density of cells varies as a function of distance from a given reference cell.

We calculated the pair distribution function for each β-cell graph in the Control and T2D groups. The distributions for large and small islets were calculated separately, since architectural differences exist between them as previously shown in [21, 23, 24]. Bounding boxes of each islet were found and used for the islet area in the calculation. The pair distribution function was then averaged for each radius over all islets in the control and T2D datasets.

We also examined the pair distribution functions for other cell types found in islets, namely α-α, α-β, α-δ, β-δ, and δ-δ. For distributions of the same cell type, the formula above was used. For edges between vertices of mixed cell types (say, cell types *a* and *b*), a different version of *g*(*r*) was used to capture the distribution of *b* with respect to *a*, $g_{ab}\left(r\right)dr=\frac{IsletArea}{2\pi rN_{a}N_{b}}\sum_{i=1}^{N_{a}}\sum_{j=1}^{N_{b}}\delta \left(r-r_{ij}\right)$ where *N*_*a*_ and *N*_*b*_ are the number of *a* and *b* vertices, respectively.

### Graph measures

Human islets have been described with a variety of qualitative forms: α-β cell core subunits [25], lobules [26], cloverleaf patterns [24], ribbon-like structures [27], and folded trilaminar plates [23]. Our aim was to characterize the structure of islets in an alternate form, amenable to quantitative analysis in a static or a stochastic Langevin approach. The structure of graphs can be characterized quantitatively in a variety of ways. Here we define the most basic graph measures: the mean degree of vertices, the mean number of components per graph, and the mean number of vertices per component.

The measures we consider (degree, component size and cells per component) are amongst the simplest numbers associated with a graph. We found (see Results) that these were already biologically interesting as they could distinguish between T2D and normal islets. One measure we decided against using was the number of cliques, defined as a subgraph such that every vertex in the subgraph shares an edge with every other vertex in the subgraph. We found preserving the number of cliques to be too strong a constraint to maintain with random rearrangements.

### Mean degree

The degree, *d*_*v*_, of vertex *v* is the number of edges containing *v* as a vertex (Fig 1G). For islet graphs, this measures the number of β cells in direct contact with a given β cell. To define *d*_*v*_, let *E*(*a*,*b*) = 1 if *e*_*ab*_, the edge between vertices *a* and *b*, exists and *E*(*a*,*b*) = 0 otherwise. Then $d_{v_{a}}=\sum_{i=1}^{n}E\left(a,i\right)$ where *n* is the total number of vertices in the graph. Thus, the mean degree can be defined as $\frac{1}{N}\sum_{j=1}^{N}d_{v_{j}}=\frac{1}{N}\sum_{j=1}^{N}\sum_{i=1}^{n}E\left(j,i\right)$ where *N* is the number of vertices in the dataset. For a given neighborhood radius, the degree of each cell of each islet was calculated and averaged together for the control and T2D groups.

### Connected components and the mean number of cells per component

The second characteristic, the connected components, describes discrete clusters of β cells. A component of a graph is a subset of vertices contained in the graph such that each vertex in the set shares an edge with at least one other vertex in the subset (Fig 1H). That is, if a closed curve can be drawn around a subset of a particular graph without crossing an edge, and this subset contains no other smaller subsets with this property, then the subset is a component. A component can also be singular, i.e. contain only one vertex, and thus describe lone β cells. In light of this, both the singular + nonsingular components and the nonsingular-only components, which represent β-cell clusters, were examined. For a given neighborhood radius, the components of each islet were found. The number of components per islet and number of cells per component for singular + nonsingular and nonsingular-only components were then averaged over the control and T2D groups for small, large, and all (small+large) islets.

### Two-dimensional sections vs. three-dimensional volume: islet graph quantification

While islet structure images obtained from selected two-dimensional (2d) sections of pancreata have been used to support qualitative conclusions regarding islet function for decades, we wondered if graph theoretical measures deduced from 2d sections might have artifacts that would impact the validity of our graph theory approach. A systematic study of three-dimensional (3d) volume estimates from 2d is given in [51]. In other words, could it be that actual 3d islet graphs leads to graph measures that are very different from the graph measures deduced from 2d sections, without invalidating all the conclusions in the literature that are based on 2d sections alone? There are not many published human 3d islet structures to test this, but to examine the effects of studying 3d islets in 2d slices, we took a different dataset consisting of 28 islets (17,539 α cells, 32,947 β cells] that were sliced every 15 microns resulting in (*x*,*y*,*z*) coordinates where *x* and *y* represent the placement of the cell in the slice and *z* represents the height of the slice in the islet. We then perturbed the *z* value of each cell by a random number between -7.5 and 7.5 such that the cells maintained a minimal distance of 4 microns. We randomized this dataset 10 times resulting in a three-dimensional dataset that was then used for a direct comparison between resulting graph measures in 3d and in 2d after simulated sectioning.

For the 3d case, graphs were created with specified edge type and neighborhood-sphere size. For 2d case, the dataset was sliced every 15 micron throughout the volume with respect to the *z* coordinate, i.e. the volume was sliced at *z* = 0, 15, 30, …. To examine the dependency of the placement of the slice, the starting point of the slicing was shifted by a certain number of microns i.e. the volume was sliced at *z* = 1, 16, 31, … for slice-start = 1, the volume was sliced at *z* = 2, 17, 32, … for slice-start = 2 and so on for slice-starts ranging between 0 and 14. For each slice, two-dimensional graphs were created where edges were created with specified edge type and neighborhood size (based solely on the (*x*,*y*) coordinates of the cells).

### Stochastic process on graphs

To simulate stochastic graph-altering processes akin to the random walk and linear restoring force that define the Ornstein-Uhlenbeck process, we simulated vertex deletion and addition moves in each islet. We emphasize that these processes are a mathematical representation of all the developmental and homeostatic processes that resulted in the observed islet graphs. The question we focused on is: What possible graph stochastic processes would preserve the observed simple quantitative graph measures of such a set of islet graphs? Thus the deletion and addition moves do not represent actual β-cell death and birth processes, which are well-known to be rare [28], but just as random steps and a linear restoring force with appropriate parameters will preserve the histogram of a sample of normal random numbers, we aimed to find graph dependencies for vertex addition and deletion moves that would preserve the observed distributions of graph measures.

**Fig 2:**
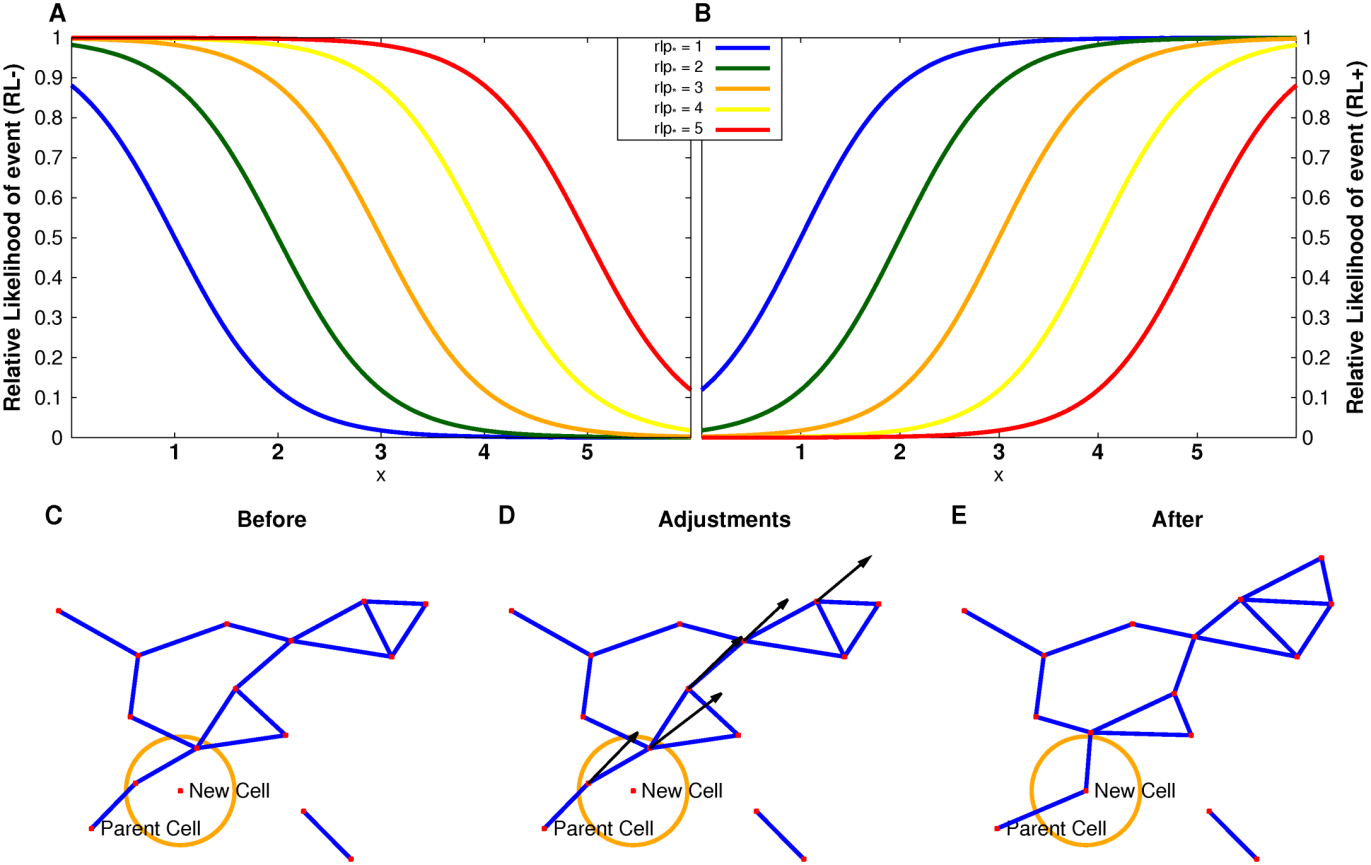
Details of Langevin model setup. Relative likelihood functions for a set of relative likelihood parameters (A,B). The function *RL*− = 0.5−0.5*tanh(*x*−rl𝑝*) for rlp* = 1…5 (A) describes the scenario where vertices with small component sizes or degrees have a higher relative likelihood of being selected for theoretical cell addition or deletion. Whereas, in the scenario with function *RL*+ = 0.5+0.5*tanh(*x*−rl𝑝*) for rl𝑝* = 1…5 (B), vertices with large component sizes or degrees have a higher relative likelihood of being selected. Cell addition (C-E). C. The new cell was added in a random direction from the parent cell which results in two cells being within the given minimal distance of the new cell. D. These cells are moved radially outward from the parent cell. This move causes other cells to be within the given minimal distance and they are in turn moved outward. E. The resulting islet after the correction.

We considered two different classes of models depending on the dependencies of the vertex addition and deletion processes. Each model was characterized by relative likelihood (*RL*) functions chosen such that cells whose graph characteristics are either above (*RL+*) or below (*RL-*) a given parameter value have a higher likelihood of an event such as the theoretical addition of a cell in its vicinity or the theoretical deletion of that cell. The sigmoidal functions are

$$RL+=0.5+0.5*\text{tanh}\left(x-rlp_{*}\right)$$ (1)

and

$$RL-=0.5-0.5*\text{tanh}\left(x-rlp_{*}\right)$$ (2)

where *x* is the graph measure defining that class of models and rlp_*_ is the addition/deletion parameter value (Fig 2A and 2B). In general, stochastic simulations with hard transitions, as opposed to the sigmoidal functions defined here, will lead to artifacts. We tried to avoid such artifacts with the use of the *RL+/-* functions. In a deterministic process, such sharp transitions for where a cell can or cannot be placed would be required, as such sharp transitions would be part of the definition of the rules for such a process. There is no evidence for such determinism in any published experimental data.

We describe each class of model in turn. Each simulation has a given *RL* function for the modeled addition (*RL*_*a*_) step with a given relative likelihood parameter (*rlp*_a_) and modeled deletion (*RL*_*d*_) step with a given relative likelihood parameter (*rlp*_*d*_). We will introduce the shorthand, M for *RL*-, P for *RL*+, a given (*RL*_*a*_, *RL*_*d*_) combination as [*RL*_*a*_] [*RL*_*d*_], and a given simulation as [*RL*_*a*_][*RL*_*d*_][*rlp*_*a*_][*rlp*_*d*_] where [*RL*_*a*_] and [*RL*_*d*_] are either *M* or *P* and [*rlp*_*a*_] and [*rlp*_*d*_] represent the given theoretical addition and deletion parameters. For example, MP01 describes the simulation where M *= RL*_*a*_, P *= RL*_*d*_, *rlp*_*a*_ = 0 and *rlp*_*d*_ = 1.

### Degree-dependent model

β cells receive cues from their environment and neighboring cells [1–3, 6, 10]. We hypothesized that the theoretical addition or deletion of a cell could be influenced by the number of β-cell contacts. Here we used the degree-dependent *RL* functions as described in Eqs 1 and 2 where *x* represents the degree of the cell. Notice that for *RL*+, cells with larger degrees have a higher relative likelihood than cells with smaller degrees of a given event (addition or deletion), whereas for *RL*-, the opposite holds.

(Fig 2A and 2B, where *x* is *deg* and rlp_*_ is *rlp*_*a*_ or *rlp*_*d*_). For combinations PP, PM, MP, and MM, 500 simulations consisting of 500 iterations of cell deletion and addition per graph for each combination of *rlp*_*a*_ = 0,…,7 (representing the observed degrees in the original architecture) crossed with *rlp*_*d*_ = 0,…,7 for each dataset (control large, control small, T2D large, and T2D small islet graphs) were run. This totaled 512,000 simulations.

### Component-dependent model

In vitro isolated β cells are known to spontaneously re-aggregate into cluster-like structures [52]. It has also been shown that β-cell clusters are necessary for pulsatile glucose stimulated insulin response in vivo [6–8],[12],[53]. Finally, the proximity of β cells to islet capillaries is essential for survival [54]. Greater β-cell distance from associated capillaries has been shown to result in β-cell disappearance during islet transplantation [55]. Here we test to see if theoretical cell addition and/or cell deletion are influenced by component size. The addition (*rlp*_*a*_) and deletion (*rlp*_*d*_) relative likelihood functions used were as described in Eqs 1 and 2 where *x* now represents the size of a cell’s component. Notice that for *RL*+, cells existing within smaller components have a higher relative likelihood than cells within larger components of a given event (addition or deletion), whereas for *RL*-, the opposite holds.

For each combination of PP, PM, MP, and MM, 500 simulations consisting of 500 iterations of cell deletion and addition per graph for each combination of *rlp*_*a*_ = 1,…,5 (representing the overwhelming majority of component sizes in the original architecture) crossed with *rlp*_*d*_ = 1,…,5 for each dataset (control large, control small, T2D large, and T2D small islet graphs) were run. This totaled 200,000 simulations.

### Vertex rearrangements

We implemented methods for stochastic changes to graphs consistent with the dataset. For each iteration, one vertex was deleted from and added to each graph based on a given probability function for the theoretical deletion and addition processes. We emphasize again that these are theoretical abstractions of cell rearrangements, not to be conflated with β-cell birth and death. In particular, note that these theoretical processes are balanced in that they preserve cell number in each islet at each step in the simulation. Obviously no biologically valid birth and death simulation could coordinate the preservation of cell number. Such a rearrangement is not a trivial process because the islet graphs represent finite-sized cells with steric constraints, not idealized mathematical points. Particular care must be taken when a β-cell is moved from one component in an islet to another, because there are geometric constraints that need to be preserved for the cell-cell contact interpretation of edges in the resulting graph. The process of removing a vertex from a graph is straightforward. However, adding a vertex to a graph can cause geometric complications, especially since there is a minimal distance between two vertices that needs to be maintained. The addition process used is: (*i*) for the chosen vertex, *v*_*parent*_, a new vertex, *v*_*new*_, is added at an angle chosen randomly from angles not occupied by a neighbor of *v*_*parent*_ and a random distance between 8 and 13 microns; (*ii*) a ‘frozen’ list is created consisting of *v*_*parent*_ and *v*_*new*_; (*iii*) a ‘problem’ list is created consisting of vertices within *d*_*min*_ distance from *v*_*new*_ where *d*_*min*_ is the minimal distance observed in the islet; (*iv*) for the first vertex on the ‘problem’ list, *v*_1_, the closest vertex on the ‘frozen’ list, *v*_*c*_, is found and *v*_1_ is moved radially from *v*_*parent*_ a given distance such that the new distance between *v*_*1*_ and *v*_*c*_ is between *d*_*min*_ and 10 μm; (*v*) the vertices within *d*_*min*_ from *v*_1_ are added to the bottom of the ‘problem’ list; and (*vi*) *v*_1_ is removed from the ‘problem’ list and added to the ‘frozen’ list (*vii*) steps (*iv*) through (*vi*) are repeated with *v*_1_ representing the first vertex in the ‘problem’ list until it is empty. This algorithm intuitively envisions a propagating wave from *v*_*parent*_ in the direction of *v*_*new*_ where vertices too close to *v*_*new*_ are moved outward, and vertices that are now too close to the just-moved vertices are moved outward, and so on until the exterior vertices of the graph are reached (Fig 2C–2E).

### Computation and statistics

Graphs were created using the Boost Graph Library [56] which utilizes a depth-first search algorithm for computing components [57]. Additional code was written in C++. For simulations, this study utilized the high-performance computational capabilities of the Biowulf Linux cluster at the National Institutes of Health. The Mann-Whitney test, with a Bonferroni multiple comparison correction, was used for statistical tests.

To verify simulation convergence, the maximal standard deviation and maximal change in mean of each graph measure were calculated with the addition of each simulation. For simulation *i*, the standard deviations of simulations 1, …,*i* were calculated for each (*rlp*_*a*_,*rlp*_*d*_). The maximal standard deviation is the maximum of these values and was found and plotted for *i* = 1…*n*, where *n* is the number of simulations. The change in mean was found with the addition of each simulation for each (*rlp*_*a*_,*rlp*_*d*_). The maximal change in mean is the maximum over all (*rlp*_*a*_,*rlp*_*d*_). For convergence, as *i* approaches *n* the maximal standard deviation approaches a constant and the maximal change in mean approaches zero.

## Results

### Islet and cellular composition

The number of islets obtained from each donor is given in Table 1, with the number of cells in large and small islets. We counted the number of endocrine cells of each type in large and small islets (Table 2). Notice the decrease in all cell types in large T2D islets as compared to control islets.

**Table 2:** Quantifications of the number of α, β, and δ cells found in large and small islets for Control (C) and T2D islets.

		Large		Small		Total	
		C	T2D	C	T2D	C	T2D
**α cells**	**# of cells**	56726	31251	6305	6916	63031	38167
	**% cells**	34.5%	38.10%	22.89%	24.11%	32.83%	34.48%
	**Avg#/islet**	19.59	16.57	1.05	1.14	7.06	4.81
**β cells**	**# of cells**	88857	42915	18594	19370	107451	62885
	**% cells**	54.04%	52.32%	67.49%	67.51%	55.97%	56.26%
	**Avg#/islet**	30.69	22.75	3.09	3.20	12.05	7.86
**δ cells**	**# of cells**	18832	7851	2651	2404	21483	10255
	**% cells**	11.45%	9.57%	9.62%	8.38%	11.19%	9.26%
	**Avg#/islet**	6.50	4.16	0.44	0.40	2.41	1.29

### Neighborhood size

**Fig 3:**
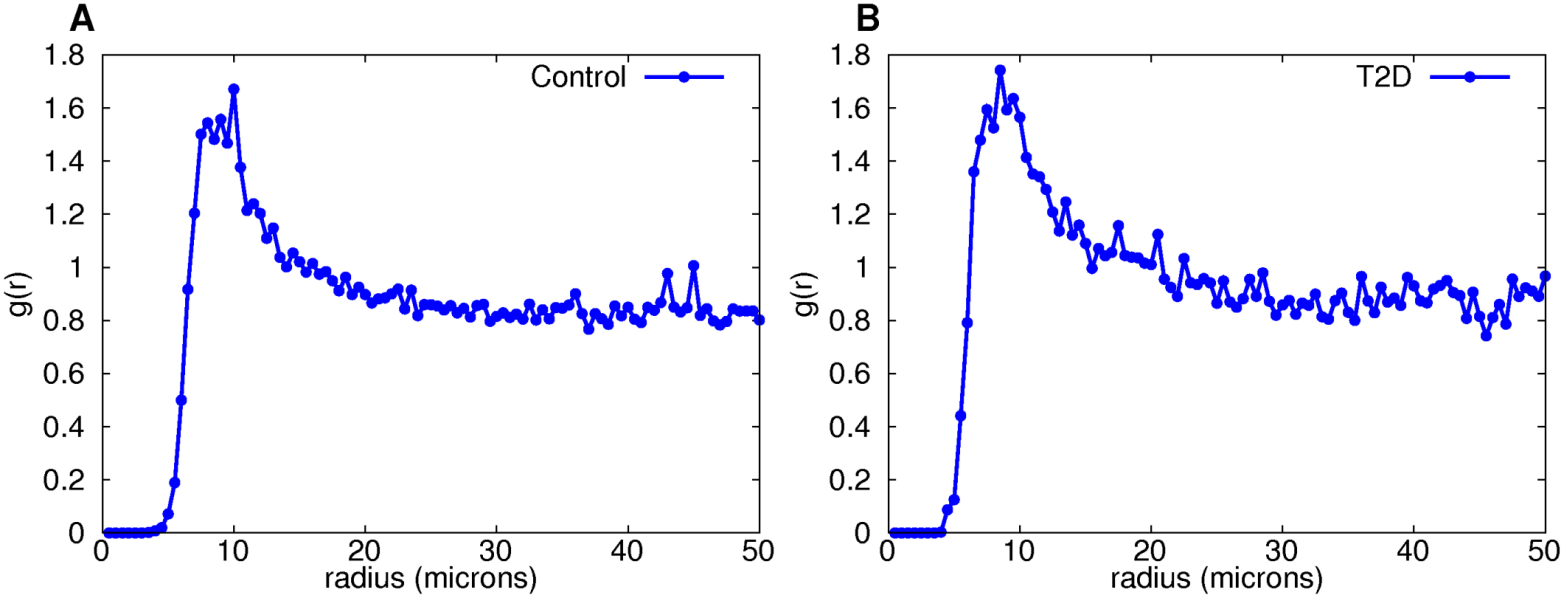
Pair distribution functions for large-islet β-cell graphs provide evidence that cell distances between 8 and 13 microns are non-random. The sole peak is clearly evident in the control (A) and diabetic (B) groups.

The pair distribution function showed a peak between 8 and 13 microns for all types of edges in large islets. Due to this, all measures were calculated for graphs with neighborhood radii between 8 and 13 (with radii 5–7 and 14–16 added for comparison). β-β edges are shown in Fig 3 with other edge types in S1–S3 Figs. Small islet pair distribution functions do not show the expected asymptotic approach to 1, suggesting that there are not enough small islets for averaging over islets to produce stable results.

### 2d vs. 3d graph measures are qualitatively the same

**Fig 4:**
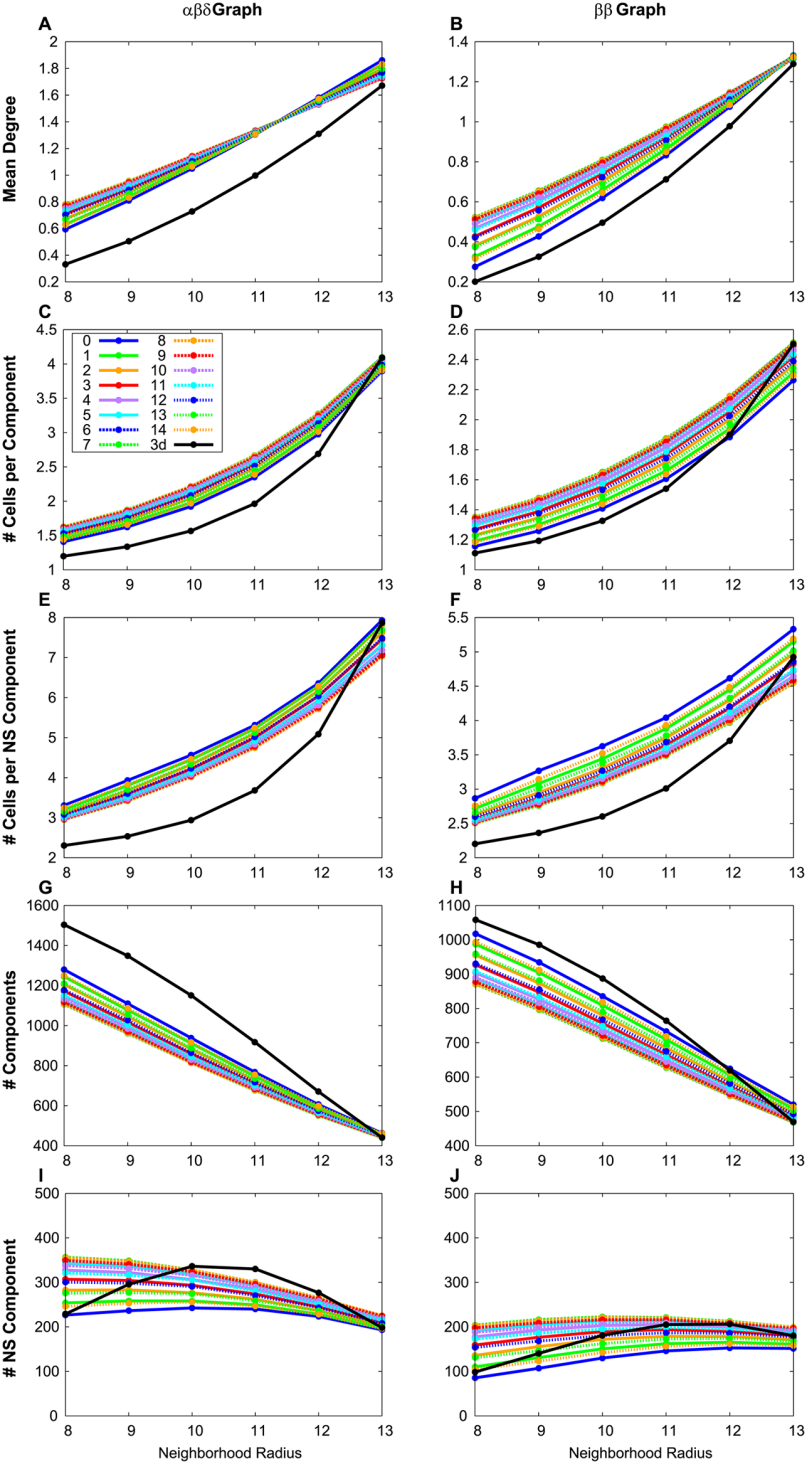
Comparison of measures between two-dimensional sections and a three-dimensional volume. The mean degree (A,B), number of cells per component (C,D) and nonsingular component (E,F), and the number of components per graph (G,H) and nonsingular components per graph (I, J) were calculated for the three-dimensional volume and the two-dimensional sections with varying slice start values (different colors) as described in the text.

We found that graph measures determined from 2d sections and 3d islet graphs are qualitatively the same for any choice of neighborhood size (Fig 4). Conclusions drawn from 2d sections should therefore be reflective of 3d differences. Of course, the significance of differences between T2D and normal sections has nothing to do with the 3d vs. 2d issue addressed here as any geometric difference would affect both types of sections alike.

### Islet graph measures can distinguish T2D from normal islets

**Fig 5:**
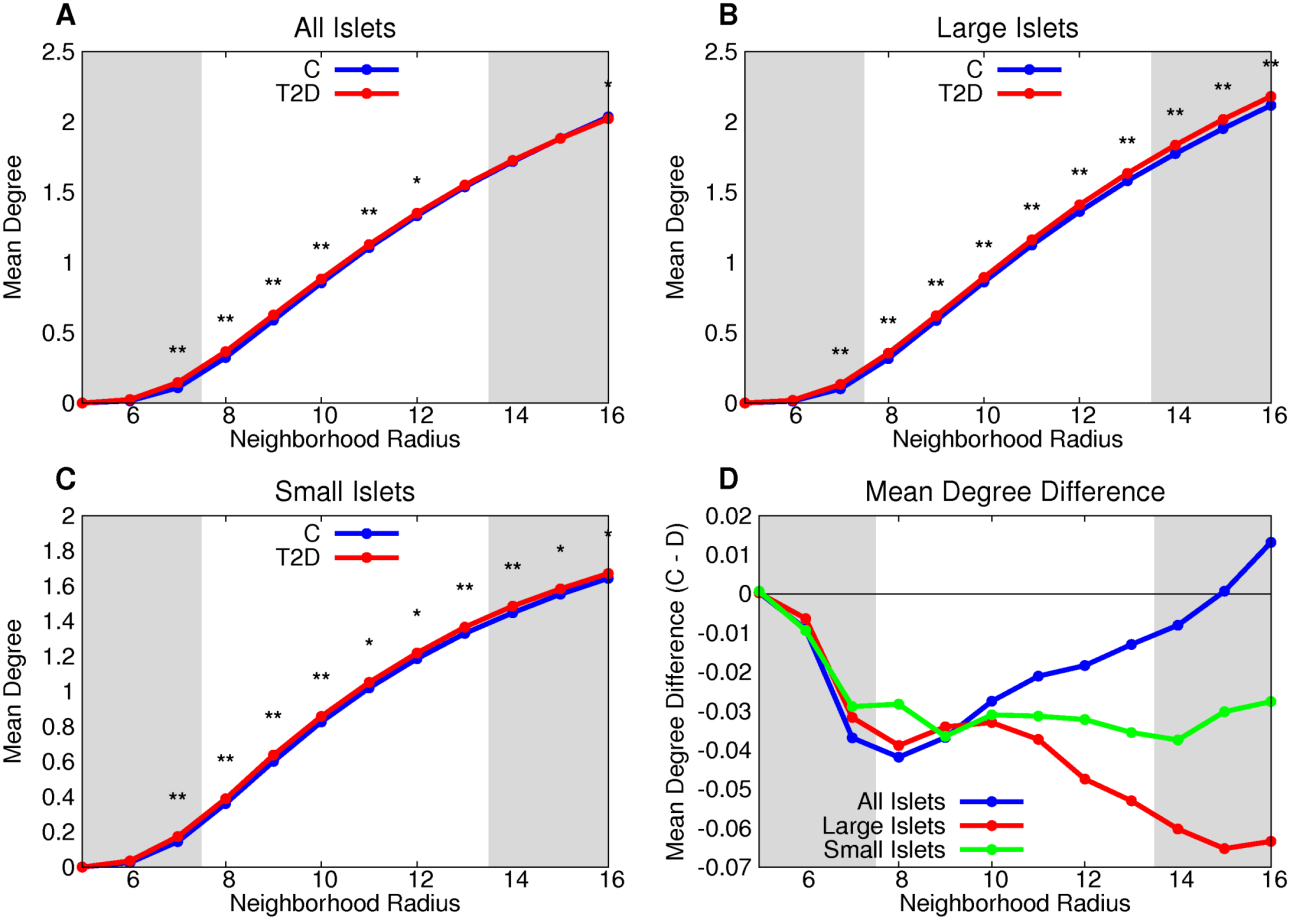
T2D β cells have, on average, more neighbors than control β cells. Both large (B) and small (C) T2D islets have a greater mean degree than control for all neighborhood radii. This is further emphasized by the difference between the two groups (D). * denotes p < 0.05 between the control and T2D groups, ** denotes a statistically-significant difference after the Bonferroni correction (using *n* = 32).

**Fig 6:**
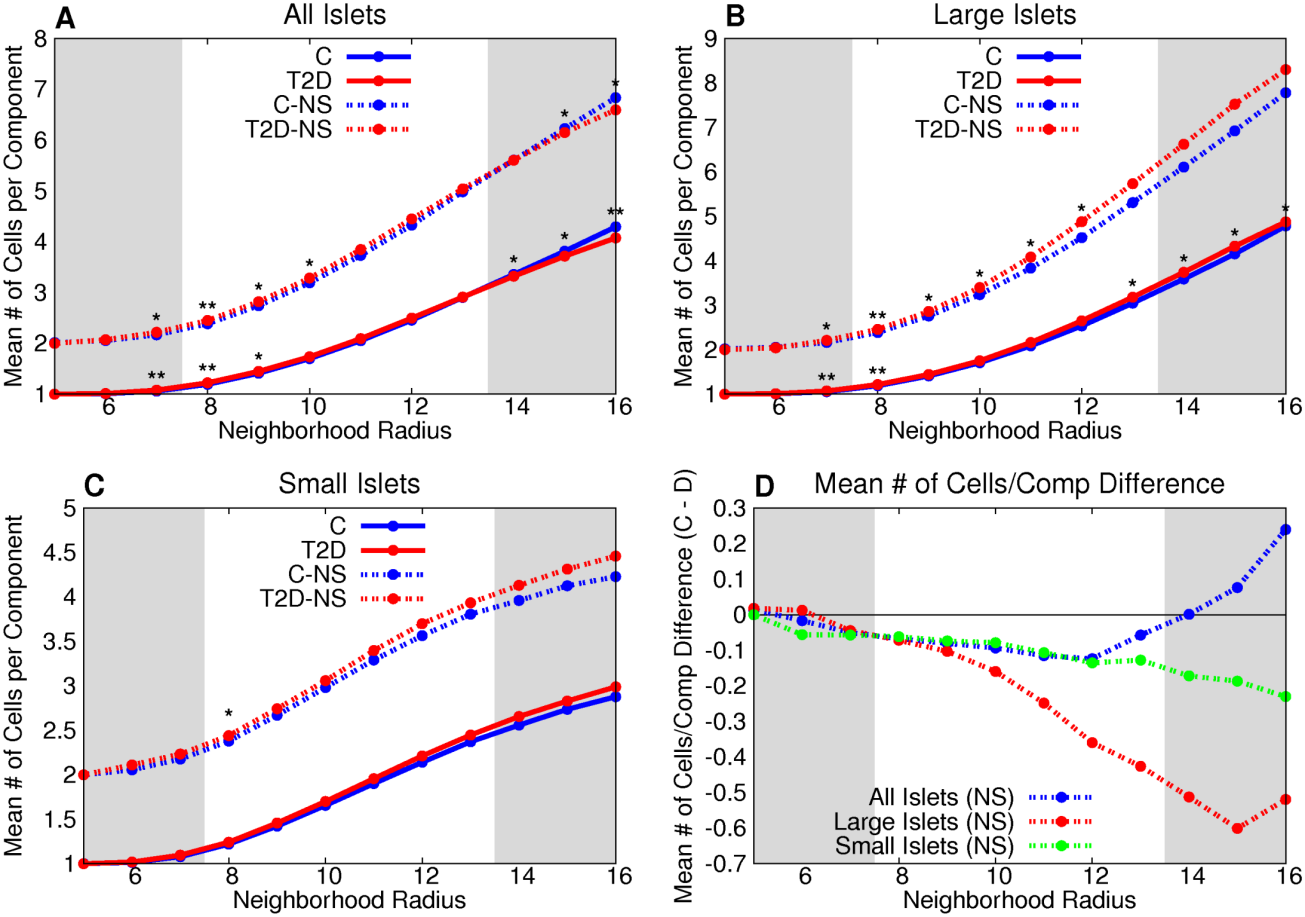
T2D β-cell clusters in large islets have, on average, more cells than control β-cell clusters in large islets. The number of vertices per non-singular component is greater in the T2D large-islet group than the control for graphs of neighborhood radii between 7 and 12 microns (B). This is further emphasized by the difference between the two groups (D). The difference between T2D and control was not statistically significant in small islets or the combination of small and large islets across the range of neighborhood radii (A,C). * denotes p < 0.05 between the control and T2D groups, ** denotes a statistically-significant difference after the Bonferroni correction (using *n* = 64).

Both large and small T2D islets have a greater mean degree than control for all neighborhood radii (Fig 5). The number of vertices per non-singular component is greater in the T2D large-islet group than control for graphs of neighborhood radii between 7 and 12 microns (Fig 6). The difference between T2D and control was not statistically significant in small islets or the combination of small and large islets across the range of neighborhood radii (Fig 6A and 6C).

**Fig 7:**
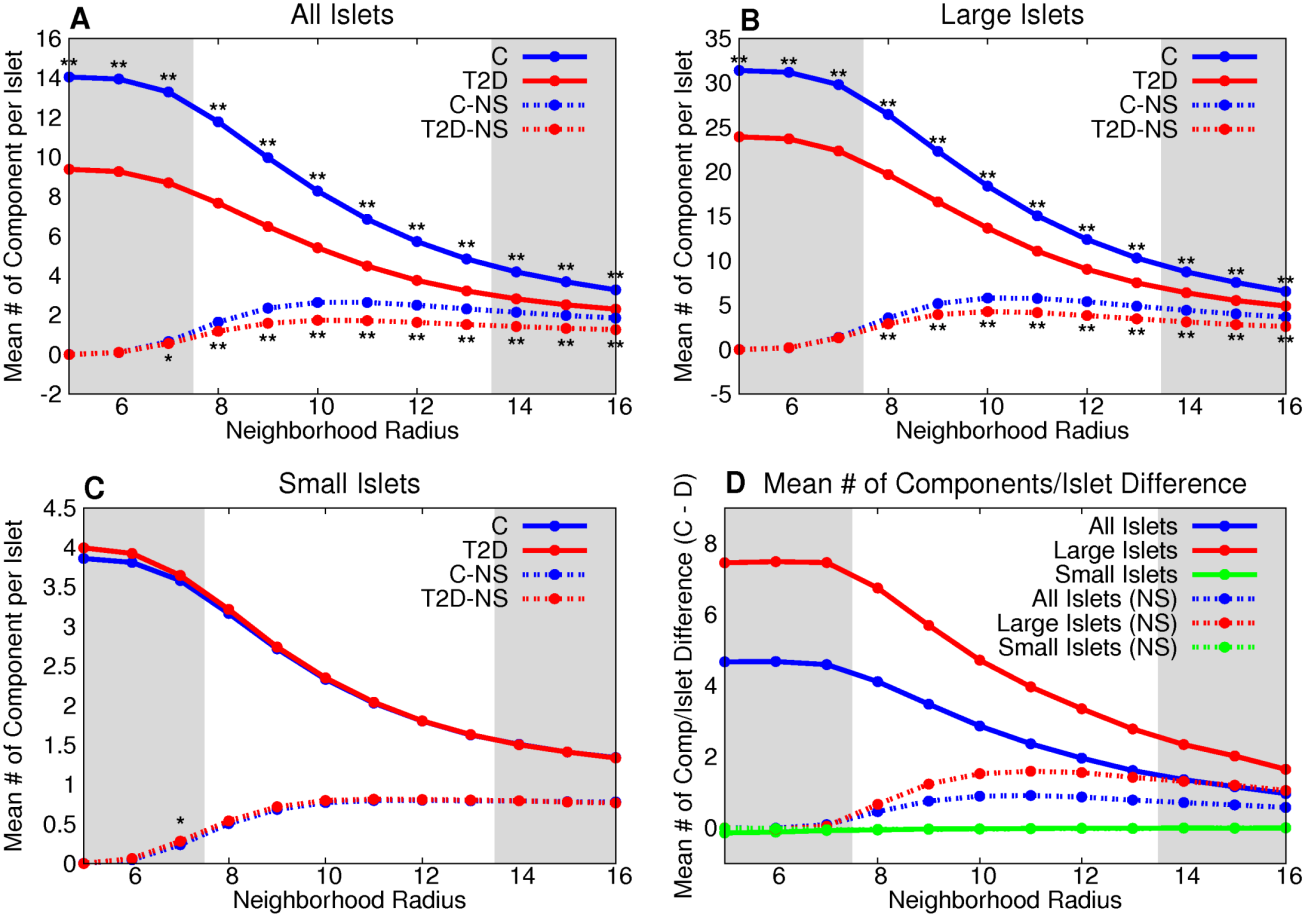
T2D large islets have fewer β-cell clusters than control large islets. The number of components per graph is significantly lower in large islet graphs (B) and when all islets are combined (A) with T2D than the control group. This is further emphasized by the difference between the two groups (D). However there is no statistically significant difference between the groups for small islets (C). * denotes p < 0.05 between the control and T2D groups, ** denotes a statistically-significant difference after the Bonferroni correction (using *n* = 64).

The number of components per graph is significantly lower in large islet graphs (Fig 7B) and when all islets are combined (Fig 7A) with T2D than the control group. There is no statistically significant difference between the groups for small islets (Fig 7C). The size dependence and cumulative distributions of the three graph measures for all, large, and small islets, respectively, are shown in S4–S6 Figs. The number of components per islet is significantly different between control and T2D groups even when comparing by subject (S4 Table, all measures were compared by subject in S1–S15 Tables).

The differences in mean degree and number of components seen in T2D islets cannot be explained by the loss of β cells alone (S7 Fig). The differences observed in T2D cannot be explained by age (S8 Fig). We also compared the number of components per islet with the mean degree of the islet controlling for the number of cells per islet. There was a statistically significant difference (P< 0.0001) between the control and T2D group. Therefore, the decrease in components observed in the T2D group as compared to the control group is not solely from the increase in mean degree. There is a difference in the mean degree and number of cells per component between large and small islets for the control and T2D datasets (S16 Table). However, this difference is dependent on the neighborhood radius.

### Stochastic graph component-dependent model on an individual islet

**Fig 8:**
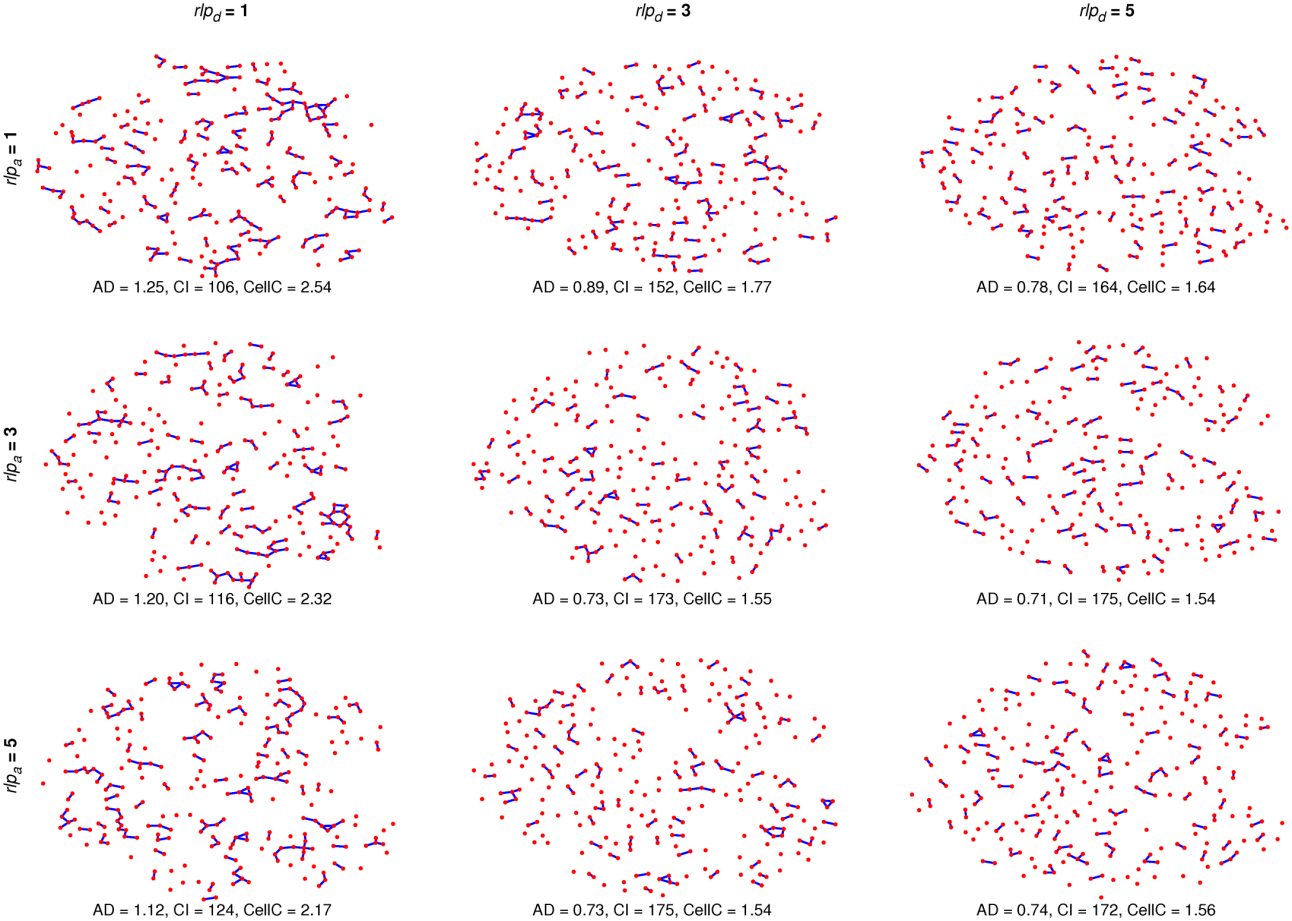
Effects of the component-dependent *rlp*_*d*_ and *rlp*_*a*_ on an individual islet. MP model simulations on an individual islet with *rlp*_*a*_ = 1, 3, 5 crossed with *rlp*_*d*_ = 1, 3, 5 were run and the resulting architectures (and measures) are shown. The original architecture’s average degree (AD) is 1.21 with 119 (46 nonsingular, 73 singular) components (CI) and an average of 2.26 cells per component (CellC).

To see the effects of different parameter values, we ran the component-dependent MP model on an individual islet with the original graph measures given in Fig 8. The resulting architectures are a mix of the islet containing varying numbers of large clusters (Fig 8, *rlp*_*d*_ = 1) and varying numbers of clusters consisting of only 2 or 3 cells (Fig 8, *rlp*_*d*_ = 5). See S1–S4 Videos for simulations of the component-dependent model using (*rlp*_*a*_,*rlp*_*d*_) = (3,1) for the PP, PM, MP, and MM models, respectively.

### Only some stochastic processes are consistent with the observed graph measures

**Fig 9:**
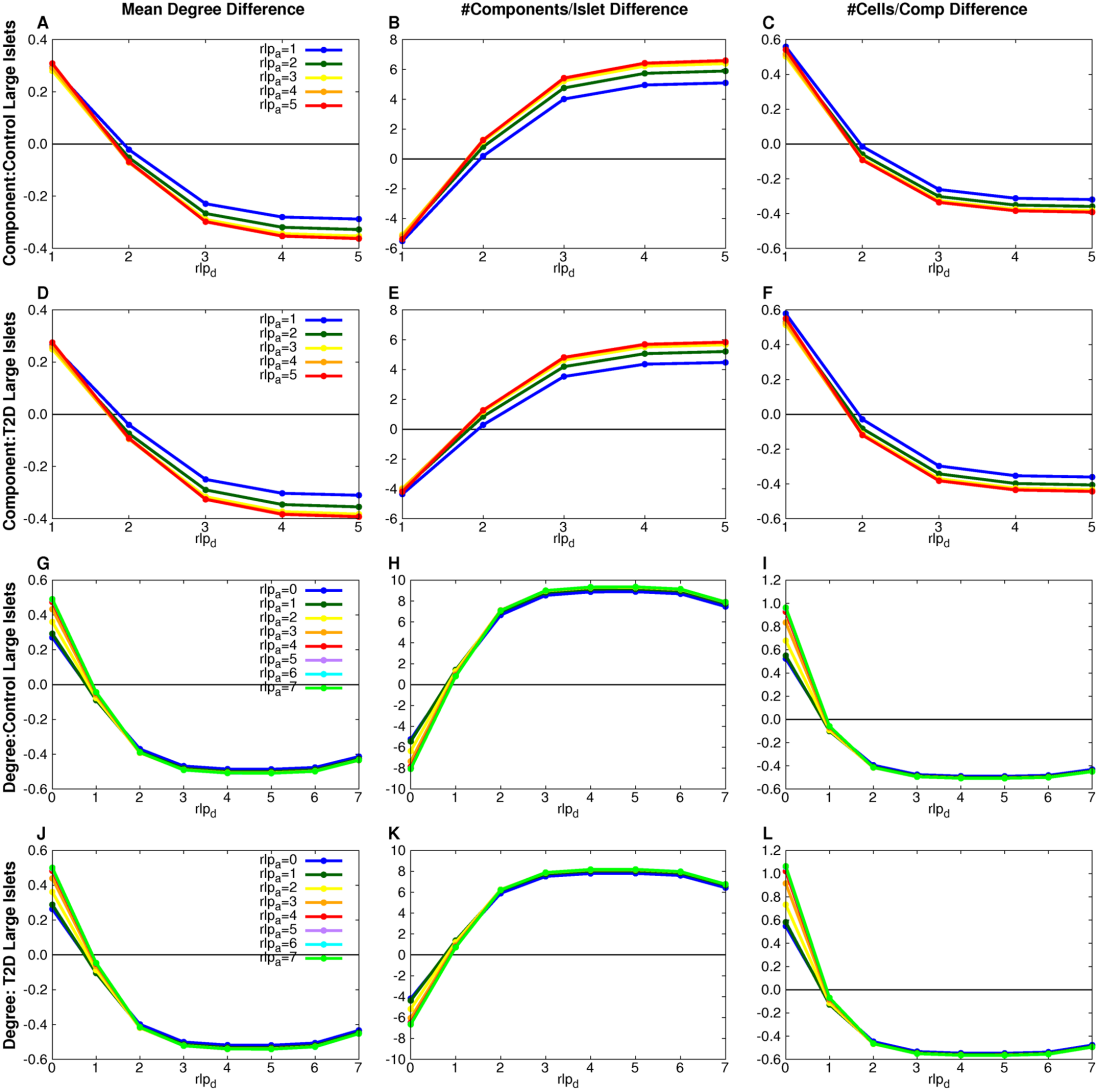
MP measure-equilibria for component- and degree-based simulations. The difference between the simulation and experimental measure values is plotted for each (*rlp*_*a*_,*rlp*_*d*_) combination (points on curves). Measure-equilibria (where measure difference between simulation and experimental is 0) were found for different (*rlp*_*a*_, *rlp*_*d*_) values for the component-based model for the control (A-C) and T2D (D-F) large islet datasets and the degree-based model for the control (G-I) and T2D (J-L) large islet datasets.

**Fig 10:**
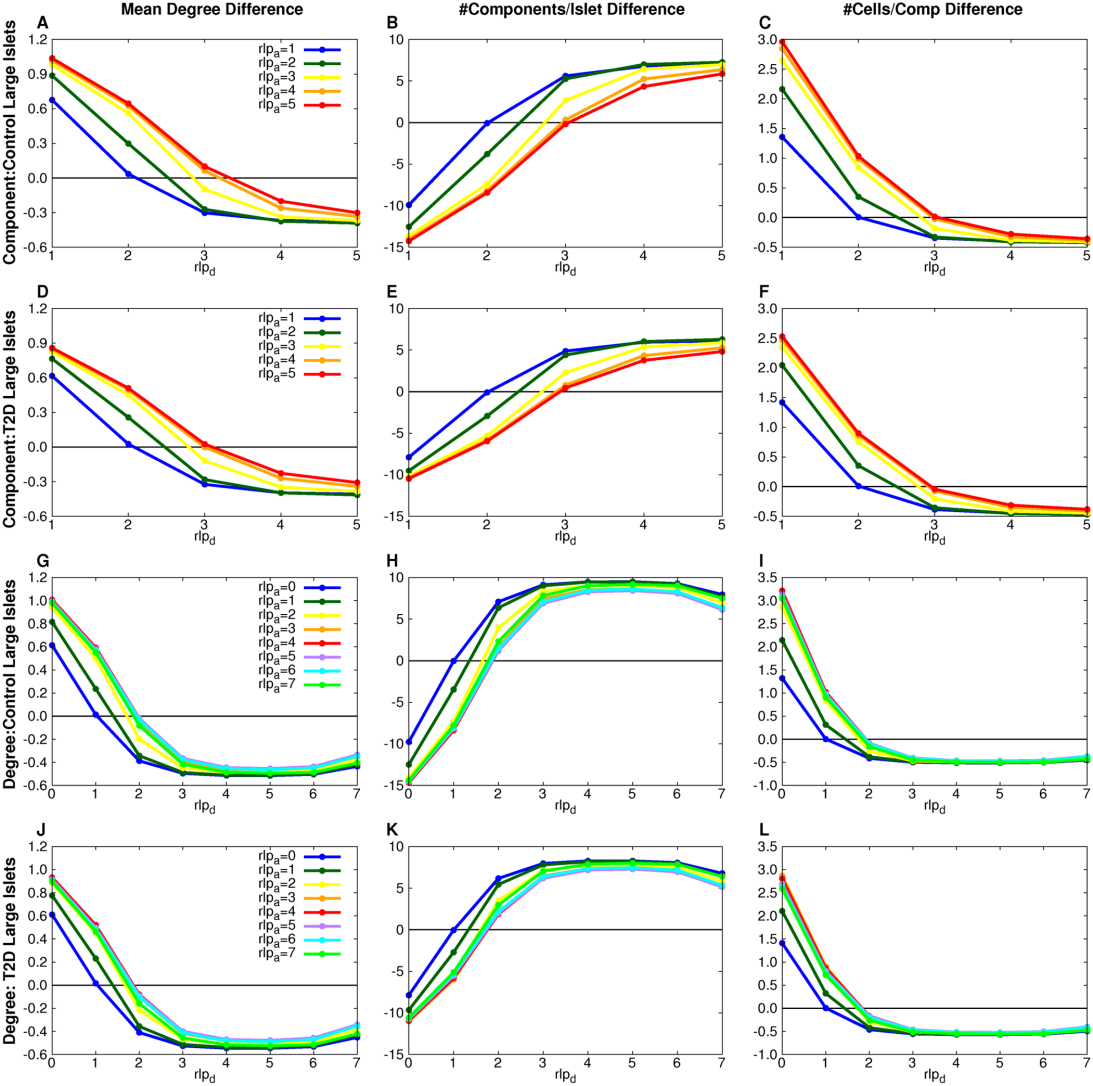
PP measure-equilibria for component- and degree-based simulations. The difference between the simulation and experimental measure values is plotted for each (*rlp*_*a*_,*rlp*_*d*_) combination (points on curves). Measure-equilibria (where measure difference between simulation and experimental is 0) were found for different (*rlp*_*a*_, *rlp*_*d*_) values for the component-based model for the control (A-C) and T2D (D-F) large islet datasets and the degree-based model for the control (G-I) and T2D (J-L) large islet datasets.

Table 3 shows all the stochastic graph processes with parameter ranges in which they are consistent with the observed graph measures. Random theoretical addition and deletion processes do not preserve the graph measures found for the experimental data (S17 Table). There are two solutions possible for both degree-based and component-based models, for both normal and T2D islets. The solutions have different characteristics. The solutions that have precise parameter values are those in which cell deletion is more likely to occur from larger components (degrees) while cell addition is unconstrained by component size (degree). These solutions are termed MP solutions (Fig 9). The other solutions are PP solutions in which both cell deletion and cell addition have a range of correlated values (Fig 10). Notice that the cell deletion parameter decreases as the cell addition parameter decreases, an intuitively natural compensation. These PP models (degree- or component-based) are therefore, mathematically-speaking, over-parametrized, but that is not a biologically valid reason for rejecting them.

**Table 3:** Mean measure-equilibria for control and T2D, large and small islets. Ranges of equilibrium solutions, with respect to *rlp*_*d*_, are given for the degree (D), number of components per islet (Comp), and number of cells per component (Cell) for PP and MP. The measure equilibria given are the *x*-intercepts of quadratic interpolations of each *rlp*_*a*_ curve. The mean represents the mean value over all equilibria over all measures for each scenario. There were no equilibrium solutions found for PM and MM.

				Control Large		Control Small		T2D Large		T2D Small	
	*RL*_*a*_	*RL*_*d*_		Measure Range	Mean	Measure Range	Mean	Measure Range	Mean	Measure Range	Mean
**Component–based Simulations**	P	P	D	2.16–3.42	2.76	1.09–1.28	1.18	2.10–3.21	2.68	1.06–1.28	1.15
			Comp	2.07–3.15		1.05–1.20		2.04–3.01		1.01–1.16	
			Cell	2.29–3.05		1.05–1.22		2.27–2.99		1.00–1.18	
	P	M		-		-		-		-	
	M	P	D	1.79–1.93	1.89	0.85–0.91	0.90	1.70–1.84	1.82	0.78–0.85	0.81
			Comp	1.79–1.97		0.91–0.95		1.73–1.92		0.82–0.87	
			Cell	1.94–2.11		0.90–0.93		1.89–2.06		0.77–0.82	
	M	M		-		-		-		-	
**Degree-based Simulations**	P	P	D	1.21–2.11	1.95	0.003–0.45	0.26	1.18–1.97	1.83	-0.03–0.44	0.22
			Comp	1.13–1.92		-0.02–0.32		1.09–1.76		-0.07–0.28	
			Cell	1.64–2.47		-0.07–0.36		1.61–2.33		-0.15–0.31	
	P	M		-		-		-		-	
	M	P	D	0.75–1.06	1.05	-0.22–-0.07	-0.13	0.70–1.03	1.02	-0.30–-0.11	-0.21
			Comp	0.78–1.00		-0.17–-0.07		0.73–0.97		-0.25–-0.13	
			Cell	1.06–1.42		-0.27–-0.13		1.01–1.41		-0.39–-0.22	
	M	M		-		-		-		-	

**Fig 11:**
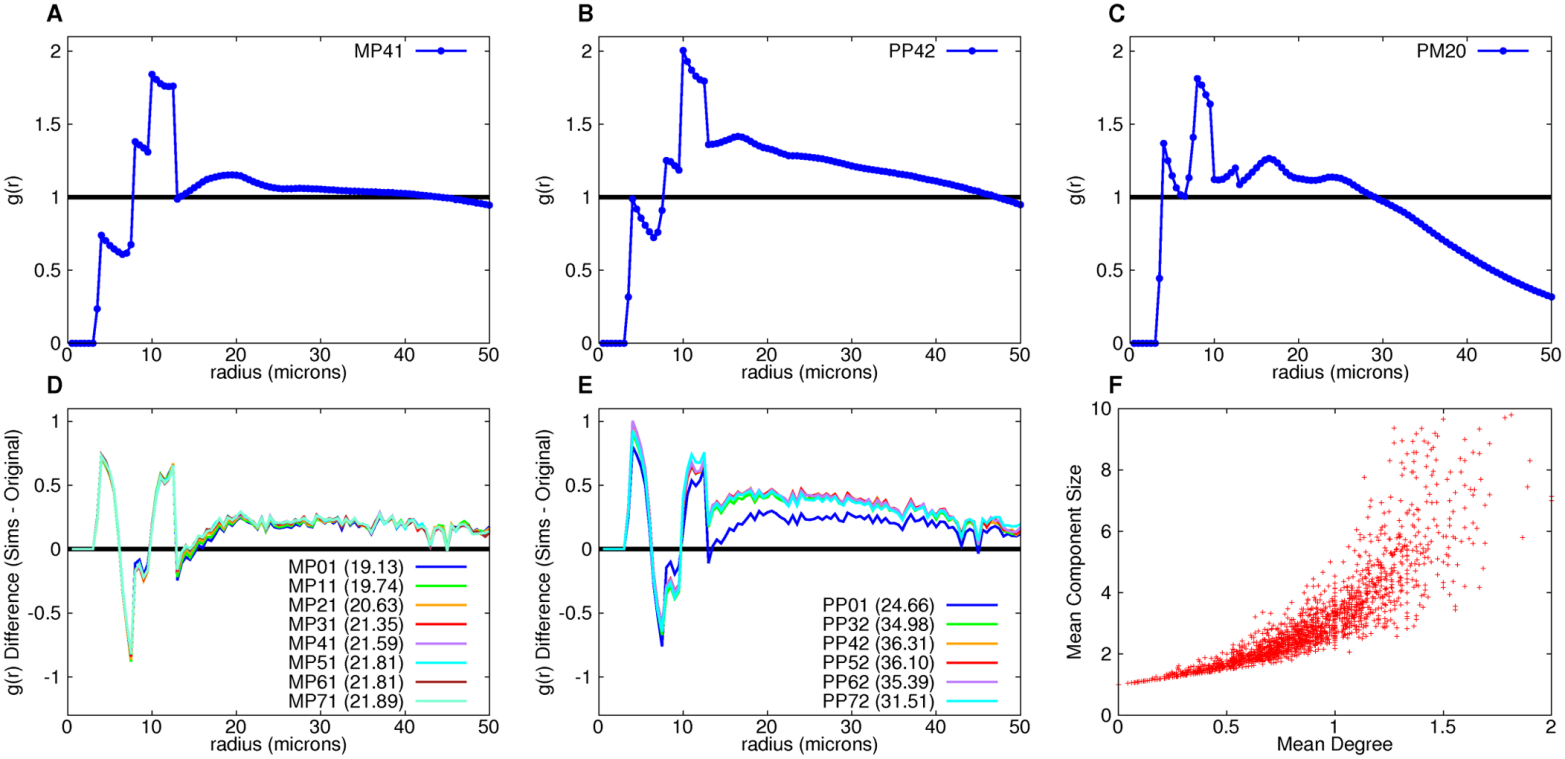
Analysis of simulation pair distribution functions. Average pair distribution functions for measure-equilibrium solutions MP41 (A) and PP42 (B) and non-measure-equilibrium solution PM20 (C). D and E. Difference between the given measure equilibrium solutions pair distributions and that of the original for given radii (cumulative total difference is given in parentheses for each parameter set). F. Mean component size versus mean degree of each control large islet.

We computed the correlation between mean degree and mean component size for each islet. We found that these are correlated (Fig 11F). Thus the degree-based and component-based solutions we found are qualitatively the same process.

All results for the component-based and degree-based models for small and large islets can be found in S9–S16 Figs. The maximal mean difference and maximal standard deviation (calculated as described in Materials and Methods-Computation and Statistics subsection) for each model is given in S17–S24 Figs, respectively.

### Pair distribution functions are qualitatively unchanged only in equilibrium solutions

Not only is it important for the resulting simulations to exhibit the original architecture’s measures, but their edge distributions should be similar as well. For example, the pair distribution functions averaged over all degree-based simulations were calculated for measure-equilibrium models MP41 and PP42 and for non-measure-equilibrium model PM20 (Fig 11A–11C). The measure-equilibrium models show two joined peaks of varying heights between edge lengths 8 and 13, which correlates with the peak found in the pair distribution of control large islets. However, the PP model distribution has values greater than 1 for radii greater than 13, whereas the non-measure-equilibrium model distribution shows multiple peaks ranging from a distance of 4 to 20, and then decays after 25 microns. To quantify the differences found when comparing the pair distribution functions for MP and PP simulations with the experimental pair distribution functions, we subtracted the difference for each radius for several MP and PP measure-equilibrium solutions (averaged over 10 simulations each) (Fig 11D and 11E) and integrated the absolute value of the difference between radius 0 and radius 50 (Fig 11D and 11E, values in parentheses). There is a noticeable increase in the total difference for the PP models. There is also an increase in total difference for non-measure-equilibrium solutions, such as MPX2 and MPX3 (S25A and S25B Fig) where X = 0–7. This is most evident within the radial distance interval of 8 and 13 where the total difference for MPX2 averaged over X = 0–7 is 3.42 and for MPX3 is 3.96 as compared to the measure-equilibrium solution set, MPX1, which is 1.88. The model with the closest match to the pair distribution function found in the control and T2D (S25C Fig) data is MP01.

The distributions of clusters (components) and the distributions for mean degree and number of cells per component for MP01 (MP02 and PP01, two other measure-equilibrium solutions, are given for comparison) simulations are given in S26 Fig. The experimentally observed distribution (S5 Fig) is qualitatively similar to that of the equilibrium solution MP01.

## Discussion

Our main descriptive result is that T2D islets have (statistically significant) quantitative differences compared to control with respect to their connectivity structure as quantified with basic graph theory measures. These subjects were being treated for T2D at the time of death, and diabetes was not the cause of death in any case. It is likely that the usual natural history of T2D progression (initial β-cell mass increase [31, 32] followed by β-cell mass loss [36] and functional loss from glucolipotoxicity [58] and inflammation [39]) has resulted in the differences we quantified. However, unlike animal models, it is not possible to attribute these changes solely to prolonged hyperglycemia and dyslipidemia.

It is striking that large T2D islets exhibit higher mean degrees, larger components but fewer components compared to control. Did the process of developing diabetes lead to the loss of cells that were not sufficiently connected, and the loss of components that were not large enough for the synergistic functional couplings between β cells needed to sustain them? Indeed, prolonged hyperglycemia, with concomitant higher demands for insulin secretion, leads to a decrease in Gjd2 expression (which codes for Connexin36 gap junction channels) in mice [59]. Further, Cx36 plays a protective role against apoptosis in the presence of toxins [18] suggesting β cells with fewer connections have less apoptotic protection. A greater number of cells in contact could be a compensation for fewer gap junction channels between each pair of cells in contact, resulting in increased degree and cells per component.

Our central deductive result from the simulations of stochastic processes on islet architectures is that it is possible to find a stochastic process that maintains the graphical measures observed with a slight difference between parameters in T2D subjects compared to control. Surprisingly, the data could rule out entire classes of stochastic rearrangement processes as implausible because of a failure to reproduce the observed architectures of islets for control and T2D. Our results indicate that β cells are preferentially moved from larger β-cell clusters while their destination clusters are picked randomly.

The idea here was not to examine changes in topology due to change in β-cell mass, as that would require having information about the entire natural history of progression to T2D via insulin resistance. Such information would be very valuable but does not exist at this time. In addition, the specimens were obtained from T2D donors with unknown therapeutic interventions, so the T2D dataset reflects possibly stable but inadequate β-cell mass. Therefore, we took each dataset separately and found theoretical cellular rearrangement processes that conserved the topology of each dataset as determined by the three graph measures and the resulting pair distribution functions. The distribution of theoretical clusters was essentially unchanged under the stochastic rearrangement process.

One point of interest is that the structural line between T2D and normal islets appears to be fairly fine, i.e., the parameter difference between the stochastic process for T2D islets and that for control is small. Whether these differences are a reflection of the compensatory processes that took place during the stages of insulin resistance preceding the appearance of T2D, or are reflective of ongoing continuing pathological changes, cannot be ascertained from such donor data. Furthermore, the limited number of 3d islets that we had to test our qualitative similarity between 3d and 2d graph measure trends does not quantitatively guarantee that a future dataset with 3d structures analyzed with 3d graph measures will find the same significant difference between T2D and control 2d graph measures. In other words, we cannot preclude that 3d islet structures analyzed with 3d graph measures could conceivably find that T2D islets are not distinct from control.

Our use of stochastic methods is motivated by two factors: (1) The exact processes of development and homeostasis in the human pancreas are unknown. Imposing a deterministic process would lead to many graph structures that might be artifacts of the simulation, as such a process would have to depend on many more parameters to detail how cells are placed depending on cell vicinity at each step. We do not know of any extant dataset that could possibly be used to constrain such deterministic simulations, nor do we know any literature on experimental results about in vivo endocrine development in the pancreas that could inform the design of an algorithm for deterministically re-arranging cells. (2) When we calculate pair distribution functions from the experimental data, it is striking that there is no evidence of systematic higher order structures, as would be hard to avoid in a deterministic process. Thus, while we cannot conclude that the actual natural history of islet development in the pancreas is stochastic, there is no experimental evidence that the placement of cells within each islet is deterministic.

The invariance of graphical measures turned out to be a constraint strong enough to determine a unique set of stochastic graph rearrangement models. There was really only one true solution since degree- and component-models are related (Fig 11F) and MP01 fits the pair distribution function best. This is a prediction that may be testable with lineage tracing techniques in animal models. Obviously, our mathematical formulation of cell rearrangements is not based on experimental data, but the equilibrium model pair distribution still captures the observed features of the pair distribution function observed in the data, and away from equilibrium, does not. This gives us some confidence that the mathematical formulation may not be too far from reality as it has been shown that endocrine cell proliferation and migration processes are not disjoint [43]. Furthermore, when we repeated the analysis on 10% of the data, we found the same results as when we did the analysis on 100% of the data, with no change in parameters of the stochastic process. This can be interpreted as a 90% check on our initial 10% model.

Among the limitations of our analysis, the most important is the lack of information on vasculature. The importance of vasculature in islets as endocrine organs needs no repetition. While it would be very interesting to carry out a more elaborate version of our simple graph theoretic simulations with data including vascularization, this would require a very large amount of 3d data as 2d sections are unlikely to capture the essential connectivity of capillaries [60].

Another limitation is that the donor characteristics available were not very detailed. With enough donor data at all age groups, it would be of great interest to follow the changes in graph measures with age, and ideally, with insulin resistance. That level of data may make it possible to consider independent addition and deletion processes to take β-cell number changes into account.

While we only addressed the β-cell contacts in this work, the proximity of α cells to β cells may be important for the suppression of glucagon secretion by insulin [61]. It would be interesting to carry out our analysis with graphs including all different endocrine cells as vertices. More data would probably be required to constrain such a model as there are many more possible rearrangements that one would need to consider.

What might be the biological rationale for the MP01 model that is preferred by our theoretical study? As we have no data on enervation and vascularization relative to the cell coordinates in the image dataset, we do not have a concrete basis for suggesting that some unknown functionally optimal amount of contact with neurons or capillaries is the cause. The functional characteristics of the sub-unit structures that Bonner-Weir and co-workers have suggested in islets [24] may provide a clue as to why β-cell migration may proceed from larger clusters to smaller.

A very challenging extension of this work would be to type 1 diabetes, where the auto-immune destruction of islets is an ongoing process. This would require donor data with duration of disease besides more specific donor characteristics. The setup of stochastic simulations to take the duration of disease into account would require considerable thought, and there would likely be dependence on the age of the donor, besides the duration of disease.

## Supporting Information

S1 Fig Pair distribution functions for αα (A-D) and αβ (E-H) graphs in large and small control and T2D islets. The sole non-random peak between 8 and 13 microns is clearly evident in the αα large-islet graphs (A,C). For small islets (B,D), the individual islets have differing peaks above one, however when averaged together the averaged values are below one. The amplitude of the peak in the distribution of the large-islet αβ graph (E,G) is smaller than the large-islet αα graph (A,C). This could be from β cells located in the interior of a cluster away from α cells. The small islets αβ pair distribution shows numerous non-random peaks of different edge lengths (F,H).
(EPS)

S2 Fig Pair distribution functions for αδ (A-D) and ββ (E-H) graphs in large and small control and T2D islets. The pair distribution functions of the large and small αδ graphs show scattered non-random peaks (A-D). The sole non-random peak between 8 and 13 microns is clearly evident in the ββ large-islet graphs (E, G). However, this is not evident in the small islets (F,H).
(EPS)

S3 Fig Pair distribution functions for βδ (A-D) and δδ (E-H) graphs in large and small control and T2D islets. The averaged pair distribution functions of the large βδ control graphs (A) shows a low amplitude peak between 8 and 13 microns, whereas the T2D graphs (C) show multiple low-amplitude peaks of varying edge lengths. The small-islet βδ graphs (B,D) show multiple non-random peaks of various edge lengths. The sole non-random peak between 8 and 13 microns is clearly evident in the δδ large-islet graphs (E, G). However, this is not evident in the small islets (F,H).
(EPS)

S4 Fig Distributions of measures for all islets, large islets and small islets for neighborhood radius 9 for the control and T2D groups. Cumulative distributions are represented by lines.
(EPS)

S5 Fig Distributions of measures for all islets, large islets and small islets for neighborhood radius 10 for the control and T2D groups. Cumulative distributions are represented by lines.
(EPS)

S6 Fig Distributions of measures for all islets, large islets and small islets for neighborhood radius 11 for the control and T2D groups. Cumulative distributions are represented by lines.
(EPS)

S7 Fig Differences observed in T2D measures cannot be explained by the decrease in β cells alone. The slopes of the linear regressions are statistically significantly different (P < 0.0001) between the control and T2D groups for the mean degree (A) and number of components (B).
(EPS)

S8 Fig Measure results for T2D subjects are not dependent on age. (EPS)

S9 Fig Component-dependent simulation solutions for control large islets. The mean measure difference (simulation—original measure) for models PP (A-C), PM (D-F), MP (G-I), and MM (J-L) and parameter values *rlp*_*a*_ = 1,…,5 crossed with *rlp*_*d*_ = 1,…,5. Equilibrium solutions (when difference is zero) are found for scenarios where vertices are removed from large components.
(EPS)

S10 Fig Component-dependent simulation solutions for T2D large islets. The mean measure difference (simulation—original measure) for models PP (A-C), PM (D-F), MP (G-I), and MM (J-L) and parameter values *rlp*_*a*_ = 1,…,5 crossed with *rlp*_*d*_ = 1,…,5. Equilibrium solutions (when difference is zero) are found for scenarios where vertices are removed from large components.
(EPS)

S11 Fig Degree-dependent simulation solutions for control large islets. The mean measure difference (simulation—original measure) for models PP (A-C), PM (D-F), MP (G-I), and MM (J-L) and parameter values *rlp*_*a*_ = 0,…,7 crossed with *rlp*_*d*_ = 0,…,7. Equilibrium solutions (when difference is zero) are found for scenarios where vertices with large degree are removed.
(EPS)

S12 Fig Degree-dependent simulation solutions for T2D large islets. The mean measure difference (simulation—original measure) for models PP (A-C), PM (D-F), MP (G-I), and MM (J-L) and parameter values *rlp*_*a*_ = 0,…,7 crossed with *rlp*_*d*_ = 0,…,7. Measure equilibria (when difference is zero) are found for scenarios where large-degreed vertices are removed.
(EPS)

S13 Fig Component-dependent simulation solutions for control small islets. The mean measure difference (simulation—original measure) for models PP (A-C), PM (D-F), MP (G-I), and MM (J-L) and parameter values *rlp*_*a*_ = 1,…,5 crossed with *rlp*_*d*_ = 1,…,5.
(EPS)

S14 Fig Component-dependent simulation solutions for T2D small islets. The mean measure difference (simulation—original measure) for models PP (A-C), PM (D-F), MP (G-I), and MM (J-L) and parameter values *rlp*_*a*_ = 1,…,5 crossed with *rlp*_*d*_ = 1,…,5.
(EPS)

S15 Fig Degree-dependent simulation solutions for control small islets. The mean measure difference (simulation—original measure) for models PP (A-C), PM (D-F), MP (G-I), and MM (J-L) and parameter values *rlp*_*a*_ = 0,…,7 crossed with *rlp*_*d*_ = 0,…,7.
(EPS)

S16 Fig Degree-dependent simulation solutions for T2D small islets. The mean measure difference (simulation—original measure) for models PP (A-C), PM (D-F), MP (G-I), and MM (J-L) and parameter values *rlp*_*a*_ = 0,…,7 crossed with *rlp*_*d*_ = 0,…,7.
(EPS)

S17 Fig Convergence measures for component-dependent simulations for control large islets (S9 Fig). Convergence measures were computed as described in Methods.
(EPS)

S18 Fig Convergence measures for component-dependent simulations for T2D large islets (S10 Fig). (EPS)

S19 Fig Convergence measures for degree-dependent simulations for control large islets (S11 Fig). (EPS)

S20 Fig Convergence measures for degree-dependent simulations for T2D large islets (S12 Fig). (EPS)

S21 Fig Convergence measures for component-dependent simulations for control small islets (S13 Fig). (EPS)

S22 Fig Convergence measures for component-dependent simulations for T2D small islets (S14 Fig). (EPS)

S23 Fig Convergence measures for degree-dependent simulations for control small islets (S15 Fig). (EPS)

S24 Fig Convergence measures for degree-dependent simulations for T2D small islets (S16 Fig). (EPS)

S25 Fig Total difference between pair distribution functions of MPX2, MPX3 and T2D MPX1 with experimental data. A. Difference between MPX2, where X = 0.7, pair distributions and that of the original for given radii. The total difference between radii 8 and 13 is 3.42. B. Difference between MPX3, where X = 0.7, pair distributions and that of the original for given radii. The total difference between radii 8 and 13 is 3.96. C. Difference between T2D MP01 and the original. (Cumulative total difference is given in parentheses for each parameter set.)
(EPS)

S26 Fig Distribution of measures for MP01, MP02, and PP01. (EPS)

S1 Table Mean degree for all islets per subject for neighborhood radii 8–13. (DOCX)

S2 Table Mean degree for large islets per subject for neighborhood radii 8–13. (DOCX)

S3 Table Mean degree for small islets per subject for neighborhood radii 8–13. (DOCX)

S4 Table Mean number of components per islet for all islets per subject for neighborhood radii 8–13. * denotes p < 0.05 between the control and T2D groups, ** denotes a statistically-significant difference after the Bonferroni correction (using *n* = 64).
(DOCX)

S5 Table Mean number of components per islet for large islets per subject for neighborhood radii 8–13. (DOCX)

S6 Table Mean number of components per islet for small islets per subject for neighborhood radii 8–13. (DOCX)

S7 Table Mean number of cells per component for all islets per subject for neighborhood radii 8–13. (DOCX)

S8 Table Mean number of cells per component for large islets per subject for neighborhood radii 8–13. (DOCX)

S9 Table Mean number of cells per component for small islets per subject for neighborhood radii 8–13. (DOCX)

S10 Table Mean number of nonsingular components per islet for all islets per subject for neighborhood radii 8–13. * denotes p < 0.05 between the control and T2D groups, ** denotes a statistically-significant difference after the Bonferroni correction (using *n* = 64).
(DOCX)

S11 Table Mean number of nonsingular components per islet for large islets per subject for neighborhood radii 8–13. (DOCX)

S12 Table Mean number of nonsingular components per islet for small islets per subject for neighborhood radii 8–13. (DOCX)

S13 Table Mean number of cells per nonsingular component for all islets per subject for neighborhood radii 8–13. (DOCX)

S14 Table Mean number of cells per nonsingular component for large islets per subject for neighborhood radii 8–13. (DOCX)

S15 Table Mean number of cells per nonsingular component for small islets per subject for neighborhood radii 8–13. (DOCX)

S16 Table Difference (Diff) of measures between large and small islets for control (C) and T2D datasets. (DOCX)

S17 Table Simulation results for randomly chosen addition and deletion processes. For each islet type, the measures were calculated for the random simulation (Sim) and compared to the experimental (Exp) measure results. The difference (Diff) between the two values is given.
(DOCX)

S1 Video Individual islet component-dependent PP31 model simulation. (GIF)

S2 Video Individual islet component-dependent PM31 model simulation. (GIF)

S3 Video Individual islet component-dependent MP31 model simulation. (GIF)

S4 Video Individual islet component-dependent MM31 model simulation. (GIF)

S1 Dataset Control and T2D data files containing the islet number, cell number, (x,y) location, and cell type for each cell in each subject. (ZIP)
