# Supplementary material for: The Beta Cell in Its Cluster: Stochastic Graphs of Beta Cell Connectivity in the Islets of Langerhans
Source: PLoS Comput Biol. 2015 Aug 12;11(8):e1004423. doi: 10.1371/journal.pcbi.1004423 (PMC4534467; doi:10.1371/journal.pcbi.1004423)
Supplement: S1 Table — (DOCX) [file pcbi.1004423.s027.docx]

|  | 8 | | 9 | | 10 | | 11 | | 12 | | 13 | |
| --- | --- | --- | --- | --- | --- | --- | --- | --- | --- | --- | --- | --- |
| Subj # | C | D | C | D | C | D | C | D | C | D | C | D |
| 1 | 0.461 | 0.289 | 0.767 | 0.562 | 1.071 | 0.828 | 1.362 | 1.109 | 1.630 | 1.393 | 1.845 | 1.644 |
| 2 | 0.274 | 0.385 | 0.526 | 0.677 | 0.766 | 0.985 | 1.005 | 1.283 | 1.227 | 1.574 | 1.437 | 1.867 |
| 3 | 0.392 | 0.462 | 0.683 | 0.721 | 0.967 | 0.975 | 1.225 | 1.210 | 1.458 | 1.418 | 1.671 | 1.612 |
| 4 | 0.270 | 0.120 | 0.500 | 0.189 | 0.739 | 0.292 | 0.962 | 0.393 | 1.171 | 0.481 | 1.357 | 0.557 |
| 5 | 0.432 | 0.192 | 0.852 | 0.331 | 1.263 | 0.444 | 1.650 | 0.551 | 2.007 | 0.640 | 2.324 | 0.730 |
| 6 | 0.180 | 0.189 | 0.317 | 0.347 | 0.472 | 0.550 | 0.650 | 0.715 | 0.811 | 0.894 | 0.979 | 1.090 |
| 7 | 0.257 | 0.428 | 0.519 | 0.772 | 0.806 | 1.124 | 1.094 | 1.484 | 1.351 | 1.817 | 1.596 | 2.121 |
| 8 | 0.213 | 0.347 | 0.482 | 0.570 | 0.770 | 0.798 | 1.075 | 1.008 | 1.369 | 1.197 | 1.660 | 1.394 |
| 9 | 0.345 | 0.263 | 0.638 | 0.472 | 0.934 | 0.690 | 1.229 | 0.906 | 1.488 | 1.096 | 1.740 | 1.271 |
| 10 | 0.420 | 0.580 | 0.705 | 0.942 | 0.989 | 1.280 | 1.235 | 1.601 | 1.446 | 1.849 | 1.652 | 2.083 |
| 11 | 0.362 | 0.497 | 0.628 | 0.793 | 0.914 | 1.075 | 1.192 | 1.326 | 1.456 | 1.575 | 1.694 | 1.801 |
| 12 | 0.260 | 0.229 | 0.484 | 0.444 | 0.700 | 0.666 | 0.905 | 0.894 | 1.120 | 1.124 | 1.320 | 1.334 |
| 13 | 0.224 |  | 0.384 |  | 0.553 |  | 0.721 |  | 0.886 |  | 1.060 |  |
| 14 | 0.257 |  | 0.372 |  | 0.469 |  | 0.575 |  | 0.677 |  | 0.772 |  |
| z-score | 0.386 | | 0.077 | | 0.026 | | 0.077 | | 0.129 | | 0.180 | |
