# Supplementary material for: The Beta Cell in Its Cluster: Stochastic Graphs of Beta Cell Connectivity in the Islets of Langerhans
Source: PLoS Comput Biol. 2015 Aug 12;11(8):e1004423. doi: 10.1371/journal.pcbi.1004423 (PMC4534467; doi:10.1371/journal.pcbi.1004423)
Supplement: S2 Table — (DOCX) [file pcbi.1004423.s028.docx]

|  | 8 | | 9 | | 10 | | 11 | | 12 | | 13 | |
| --- | --- | --- | --- | --- | --- | --- | --- | --- | --- | --- | --- | --- |
| Subj # | C | D | C | D | C | D | C | D | C | D | C | D |
| 1 | 0.438 | 0.275 | 0.741 | 0.554 | 1.050 | 0.841 | 1.371 | 1.150 | 1.656 | 1.480 | 1.889 | 1.777 |
| 2 | 0.266 | 0.404 | 0.514 | 0.726 | 0.758 | 1.075 | 1.001 | 1.417 | 1.234 | 1.744 | 1.450 | 2.092 |
| 3 | 0.379 | 0.463 | 0.677 | 0.726 | 0.971 | 1.003 | 1.239 | 1.270 | 1.488 | 1.504 | 1.721 | 1.736 |
| 4 | 0.253 | 0.099 | 0.485 | 0.167 | 0.734 | 0.273 | 0.980 | 0.370 | 1.210 | 0.459 | 1.409 | 0.541 |
| 5 | 0.428 | 0.154 | 0.850 | 0.279 | 1.261 | 0.399 | 1.650 | 0.508 | 2.009 | 0.585 | 2.327 | 0.675 |
| 6 | 0.163 | 0.172 | 0.301 | 0.324 | 0.458 | 0.545 | 0.641 | 0.714 | 0.806 | 0.914 | 0.987 | 1.119 |
| 7 | 0.236 | 0.428 | 0.499 | 0.777 | 0.800 | 1.136 | 1.100 | 1.509 | 1.368 | 1.859 | 1.631 | 2.181 |
| 8 | 0.201 | 0.333 | 0.475 | 0.556 | 0.774 | 0.785 | 1.097 | 0.998 | 1.412 | 1.204 | 1.731 | 1.411 |
| 9 | 0.336 | 0.249 | 0.634 | 0.456 | 0.939 | 0.693 | 1.252 | 0.931 | 1.528 | 1.146 | 1.795 | 1.352 |
| 10 | 0.409 | 0.555 | 0.698 | 0.915 | 0.984 | 1.263 | 1.237 | 1.608 | 1.456 | 1.873 | 1.672 | 2.121 |
| 11 | 0.354 | 0.447 | 0.620 | 0.734 | 0.908 | 1.010 | 1.195 | 1.270 | 1.466 | 1.544 | 1.713 | 1.784 |
| 12 | 0.259 | 0.218 | 0.484 | 0.430 | 0.707 | 0.662 | 0.918 | 0.912 | 1.140 | 1.169 | 1.344 | 1.391 |
| 13 | 0.212 |  | 0.371 |  | 0.535 |  | 0.709 |  | 0.879 |  | 1.060 |  |
| 14 | 0.233 |  | 0.350 |  | 0.451 |  | 0.556 |  | 0.659 |  | 0.770 |  |
| z-score | 0.283 | | 0.077 | | 0.180 | | 0.077 | | 0.129 | | 0.180 | |
