# Supplementary material for: The Beta Cell in Its Cluster: Stochastic Graphs of Beta Cell Connectivity in the Islets of Langerhans
Source: PLoS Comput Biol. 2015 Aug 12;11(8):e1004423. doi: 10.1371/journal.pcbi.1004423 (PMC4534467; doi:10.1371/journal.pcbi.1004423)
Supplement: S3 Table — (DOCX) [file pcbi.1004423.s029.docx]

|  | 8 | | 9 | | 10 | | 11 | | 12 | | 13 | |
| --- | --- | --- | --- | --- | --- | --- | --- | --- | --- | --- | --- | --- |
| Subj # | C | D | C | D | C | D | C | D | C | D | C | D |
| 1 | 0.531 | 0.307 | 0.845 | 0.572 | 1.138 | 0.813 | 1.334 | 1.056 | 1.550 | 1.283 | 1.710 | 1.476 |
| 2 | 0.321 | 0.358 | 0.594 | 0.604 | 0.809 | 0.857 | 1.026 | 1.088 | 1.191 | 1.328 | 1.362 | 1.540 |
| 3 | 0.452 | 0.462 | 0.709 | 0.713 | 0.950 | 0.928 | 1.162 | 1.106 | 1.317 | 1.272 | 1.440 | 1.400 |
| 4 | 0.320 | 0.178 | 0.544 | 0.251 | 0.755 | 0.343 | 0.909 | 0.455 | 1.060 | 0.541 | 1.207 | 0.601 |
| 5 | 0.518 | 0.248 | 0.904 | 0.409 | 1.312 | 0.512 | 1.660 | 0.616 | 1.970 | 0.722 | 2.272 | 0.813 |
| 6 | 0.238 | 0.242 | 0.370 | 0.419 | 0.517 | 0.573 | 0.677 | 0.716 | 0.829 | 0.832 | 0.953 | 0.997 |
| 7 | 0.312 | 0.428 | 0.573 | 0.739 | 0.821 | 1.044 | 1.080 | 1.306 | 1.306 | 1.523 | 1.504 | 1.710 |
| 8 | 0.255 | 0.397 | 0.507 | 0.621 | 0.755 | 0.845 | 0.997 | 1.043 | 1.220 | 1.171 | 1.412 | 1.331 |
| 9 | 0.383 | 0.287 | 0.657 | 0.500 | 0.912 | 0.685 | 1.134 | 0.863 | 1.326 | 1.010 | 1.517 | 1.131 |
| 10 | 0.460 | 0.648 | 0.734 | 1.015 | 1.010 | 1.328 | 1.225 | 1.581 | 1.406 | 1.785 | 1.572 | 1.978 |
| 11 | 0.434 | 0.567 | 0.702 | 0.875 | 0.966 | 1.166 | 1.160 | 1.404 | 1.355 | 1.618 | 1.521 | 1.823 |
| 12 | 0.273 | 0.257 | 0.495 | 0.481 | 0.622 | 0.677 | 0.760 | 0.847 | 0.891 | 1.010 | 1.033 | 1.189 |
| 13 | 0.272 |  | 0.436 |  | 0.627 |  | 0.770 |  | 0.914 |  | 1.061 |  |
| 14 | 0.322 |  | 0.430 |  | 0.517 |  | 0.624 |  | 0.725 |  | 0.779 |  |
| z-score | 0.283 | | 0.129 | | 0.129 | | 0.283 | | 0.334 | | 0.180 | |
