# Supplementary material for: The Beta Cell in Its Cluster: Stochastic Graphs of Beta Cell Connectivity in the Islets of Langerhans
Source: PLoS Comput Biol. 2015 Aug 12;11(8):e1004423. doi: 10.1371/journal.pcbi.1004423 (PMC4534467; doi:10.1371/journal.pcbi.1004423)
Supplement: S4 Table — * denotes p < 0.05 between the control and T2D groups, ** denotes a statistically-significant difference after the Bonferroni correction (using n = 64). (DOCX) [file pcbi.1004423.s030.docx]

|  | 8 | | 9 | | 10 | | 11 | | 12 | | 13 | |
| --- | --- | --- | --- | --- | --- | --- | --- | --- | --- | --- | --- | --- |
| Subj # | C | D | C | D | C | D | C | D | C | D | C | D |
| 1 | 9.78 | 6.46 | 7.98 | 5.47 | 6.36 | 4.56 | 5.09 | 3.73 | 4.13 | 3.04 | 3.55 | 2.53 |
| 2 | 11.95 | 7.42 | 10.25 | 6.15 | 8.73 | 4.91 | 7.37 | 3.88 | 6.25 | 3.07 | 5.31 | 2.41 |
| 3 | 11.58 | 6.75 | 9.60 | 5.67 | 7.84 | 4.74 | 6.44 | 3.97 | 5.34 | 3.37 | 4.52 | 2.91 |
| 4 | 7.91 | 4.58 | 6.89 | 4.41 | 5.92 | 4.18 | 5.10 | 3.94 | 4.41 | 3.74 | 3.89 | 3.58 |
| 5 | 47.42 | 3.48 | 35.37 | 3.21 | 24.99 | 3.03 | 17.25 | 2.84 | 11.94 | 2.70 | 8.58 | 2.56 |
| 6 | 7.89 | 7.39 | 7.31 | 6.77 | 6.66 | 6.01 | 5.98 | 5.40 | 5.39 | 4.79 | 4.85 | 4.27 |
| 7 | 9.35 | 17.08 | 7.97 | 13.55 | 6.57 | 10.29 | 5.31 | 7.49 | 4.38 | 5.51 | 3.64 | 4.26 |
| 8 | 12.12 | 9.23 | 10.32 | 8.05 | 8.51 | 6.92 | 6.75 | 5.96 | 5.32 | 5.19 | 4.14 | 4.54 |
| 9 | 12.14 | 5.31 | 10.07 | 4.69 | 8.17 | 4.08 | 6.52 | 3.55 | 5.29 | 3.14 | 4.31 | 2.83 |
| 10 | 10.09 | 10.71 | 8.37 | 8.22 | 6.81 | 6.28 | 5.65 | 4.76 | 4.79 | 3.86 | 4.06 | 3.25 |
| 11 | 17.73 | 8.49 | 15.03 | 6.98 | 12.34 | 5.69 | 10.03 | 4.68 | 8.14 | 3.84 | 6.77 | 3.26 |
| 12 | 15.06 | 8.77 | 13.18 | 7.72 | 11.51 | 6.70 | 10.03 | 5.72 | 8.60 | 4.79 | 7.47 | 4.07 |
| 13 | 9.90 |  | 9.04 |  | 8.16 |  | 7.34 |  | 6.61 |  | 5.87 |  |
| 14 | 4.74 |  | 4.44 |  | 4.20 |  | 3.93 |  | 3.72 |  | 3.57 |  |
| z-score | 2.597** | | 2.649** | | 2.752** | | 2.803** | | 2.906** | | 2.906** | |
