# Supplementary material for: The Beta Cell in Its Cluster: Stochastic Graphs of Beta Cell Connectivity in the Islets of Langerhans
Source: PLoS Comput Biol. 2015 Aug 12;11(8):e1004423. doi: 10.1371/journal.pcbi.1004423 (PMC4534467; doi:10.1371/journal.pcbi.1004423)
Supplement: S5 Table — (DOCX) [file pcbi.1004423.s031.docx]

|  | 8 | | 9 | | 10 | | 11 | | 12 | | 13 | |
| --- | --- | --- | --- | --- | --- | --- | --- | --- | --- | --- | --- | --- |
| Subj # | C | D | C | D | C | D | C | D | C | D | C | D |
| 1 | 25.27 | 20.90 | 20.67 | 17.65 | 16.33 | 14.49 | 12.76 | 11.48 | 10.05 | 8.94 | 8.49 | 7.03 |
| 2 | 22.38 | 28.56 | 19.24 | 23.18 | 16.34 | 17.79 | 13.75 | 13.18 | 11.54 | 9.85 | 9.73 | 6.88 |
| 3 | 28.79 | 19.10 | 23.77 | 15.97 | 19.23 | 13.11 | 15.58 | 10.61 | 12.67 | 8.71 | 10.43 | 7.13 |
| 4 | 18.21 | 8.37 | 15.84 | 8.07 | 13.52 | 7.63 | 11.45 | 7.21 | 9.77 | 6.84 | 8.50 | 6.53 |
| 5 | 75.71 | 5.58 | 56.45 | 5.20 | 39.81 | 4.87 | 27.38 | 4.57 | 18.77 | 4.39 | 13.32 | 4.17 |
| 6 | 17.92 | 17.83 | 16.59 | 16.38 | 15.09 | 14.41 | 13.50 | 12.91 | 12.15 | 11.29 | 10.84 | 9.98 |
| 7 | 24.72 | 38.36 | 21.12 | 30.30 | 17.20 | 22.78 | 13.79 | 16.18 | 11.16 | 11.48 | 9.05 | 8.52 |
| 8 | 39.81 | 18.84 | 33.85 | 16.40 | 27.69 | 14.10 | 21.55 | 12.10 | 16.44 | 10.41 | 12.19 | 9.03 |
| 9 | 27.42 | 15.16 | 22.68 | 13.41 | 18.26 | 11.53 | 14.31 | 9.89 | 11.41 | 8.57 | 9.10 | 7.55 |
| 10 | 20.56 | 26.06 | 17.03 | 20.09 | 13.83 | 15.16 | 11.37 | 11.25 | 9.52 | 8.90 | 7.93 | 7.25 |
| 11 | 32.12 | 21.76 | 27.24 | 18.03 | 22.30 | 14.79 | 17.98 | 12.05 | 14.47 | 9.62 | 11.89 | 7.92 |
| 12 | 24.25 | 21.61 | 21.23 | 19.07 | 18.44 | 16.45 | 16.00 | 13.80 | 13.62 | 11.27 | 11.79 | 9.40 |
| 13 | 20.54 |  | 18.77 |  | 16.99 |  | 15.25 |  | 13.66 |  | 12.07 |  |
| 14 | 10.01 |  | 9.39 |  | 8.88 |  | 8.32 |  | 7.90 |  | 7.51 |  |
| z-score | 1.517 | | 1.826 | | 2.032* | | 2.340* | | 2.803** | | 3.112** | |
