# Supplementary material for: The Beta Cell in Its Cluster: Stochastic Graphs of Beta Cell Connectivity in the Islets of Langerhans
Source: PLoS Comput Biol. 2015 Aug 12;11(8):e1004423. doi: 10.1371/journal.pcbi.1004423 (PMC4534467; doi:10.1371/journal.pcbi.1004423)
Supplement: S6 Table — (DOCX) [file pcbi.1004423.s032.docx]

|  | 8 | | 9 | | 10 | | 11 | | 12 | | 13 | |
| --- | --- | --- | --- | --- | --- | --- | --- | --- | --- | --- | --- | --- |
| Subj # | C | D | C | D | C | D | C | D | C | D | C | D |
| 1 | 3.243 | 3.417 | 2.638 | 2.908 | 2.157 | 2.471 | 1.854 | 2.098 | 1.638 | 1.801 | 1.470 | 1.585 |
| 2 | 3.194 | 3.625 | 2.699 | 3.101 | 2.355 | 2.601 | 2.029 | 2.209 | 1.810 | 1.856 | 1.602 | 1.614 |
| 3 | 2.960 | 3.200 | 2.502 | 2.713 | 2.130 | 2.341 | 1.865 | 2.067 | 1.679 | 1.838 | 1.552 | 1.700 |
| 4 | 2.946 | 2.000 | 2.569 | 1.920 | 2.257 | 1.833 | 2.043 | 1.717 | 1.833 | 1.630 | 1.667 | 1.572 |
| 5 | 4.781 | 2.186 | 3.603 | 1.989 | 2.658 | 1.890 | 1.986 | 1.772 | 1.644 | 1.656 | 1.438 | 1.572 |
| 6 | 2.699 | 2.552 | 2.502 | 2.304 | 2.288 | 2.096 | 2.086 | 1.912 | 1.896 | 1.776 | 1.748 | 1.624 |
| 7 | 3.481 | 3.553 | 2.959 | 2.907 | 2.511 | 2.347 | 2.081 | 1.959 | 1.787 | 1.708 | 1.580 | 1.543 |
| 8 | 3.452 | 3.172 | 2.957 | 2.779 | 2.501 | 2.393 | 2.125 | 2.082 | 1.835 | 1.898 | 1.620 | 1.705 |
| 9 | 3.625 | 2.473 | 3.039 | 2.174 | 2.550 | 1.932 | 2.173 | 1.725 | 1.881 | 1.575 | 1.640 | 1.463 |
| 10 | 3.343 | 3.962 | 2.791 | 3.004 | 2.291 | 2.381 | 1.974 | 1.908 | 1.746 | 1.640 | 1.565 | 1.494 |
| 11 | 3.270 | 4.431 | 2.770 | 3.609 | 2.336 | 2.916 | 2.031 | 2.423 | 1.783 | 2.077 | 1.615 | 1.839 |
| 12 | 2.762 | 3.443 | 2.430 | 3.011 | 2.244 | 2.657 | 2.047 | 2.371 | 1.884 | 2.109 | 1.703 | 1.863 |
| 13 | 3.106 |  | 2.829 |  | 2.509 |  | 2.296 |  | 2.102 |  | 1.917 |  |
| 14 | 1.880 |  | 1.767 |  | 1.669 |  | 1.549 |  | 1.459 |  | 1.429 |  |
| z-score | 0.129 | | 0.283 | | 0.129 | | 0.026 | | 0.026 | | 0.231 | |
