# Supplementary material for: The Beta Cell in Its Cluster: Stochastic Graphs of Beta Cell Connectivity in the Islets of Langerhans
Source: PLoS Comput Biol. 2015 Aug 12;11(8):e1004423. doi: 10.1371/journal.pcbi.1004423 (PMC4534467; doi:10.1371/journal.pcbi.1004423)
Supplement: S7 Table — (DOCX) [file pcbi.1004423.s033.docx]

|  | 8 | | 9 | | 10 | | 11 | | 12 | | 13 | |
| --- | --- | --- | --- | --- | --- | --- | --- | --- | --- | --- | --- | --- |
| Subj # | C | D | C | D | C | D | C | D | C | D | C | D |
| 1 | 1.30 | 1.17 | 1.59 | 1.38 | 1.99 | 1.65 | 2.49 | 2.02 | 3.07 | 2.48 | 3.57 | 2.98 |
| 2 | 1.16 | 1.24 | 1.35 | 1.49 | 1.58 | 1.87 | 1.88 | 2.37 | 2.21 | 2.99 | 2.61 | 3.80 |
| 3 | 1.24 | 1.30 | 1.50 | 1.54 | 1.84 | 1.85 | 2.23 | 2.20 | 2.69 | 2.60 | 3.18 | 3.01 |
| 4 | 1.16 | 1.06 | 1.33 | 1.10 | 1.54 | 1.17 | 1.79 | 1.24 | 2.07 | 1.30 | 2.35 | 1.36 |
| 5 | 1.27 | 1.11 | 1.71 | 1.20 | 2.42 | 1.27 | 3.50 | 1.36 | 5.06 | 1.43 | 7.04 | 1.50 |
| 6 | 1.10 | 1.10 | 1.19 | 1.21 | 1.30 | 1.36 | 1.45 | 1.51 | 1.61 | 1.70 | 1.79 | 1.91 |
| 7 | 1.15 | 1.27 | 1.34 | 1.60 | 1.63 | 2.11 | 2.02 | 2.90 | 2.45 | 3.94 | 2.94 | 5.10 |
| 8 | 1.12 | 1.21 | 1.31 | 1.39 | 1.59 | 1.61 | 2.01 | 1.87 | 2.55 | 2.15 | 3.28 | 2.46 |
| 9 | 1.21 | 1.15 | 1.46 | 1.30 | 1.79 | 1.50 | 2.25 | 1.72 | 2.77 | 1.95 | 3.40 | 2.16 |
| 10 | 1.26 | 1.40 | 1.52 | 1.83 | 1.87 | 2.39 | 2.25 | 3.15 | 2.66 | 3.89 | 3.14 | 4.62 |
| 11 | 1.22 | 1.33 | 1.44 | 1.61 | 1.75 | 1.98 | 2.15 | 2.41 | 2.65 | 2.93 | 3.19 | 3.45 |
| 12 | 1.15 | 1.13 | 1.31 | 1.28 | 1.50 | 1.48 | 1.72 | 1.73 | 2.01 | 2.06 | 2.31 | 2.43 |
| 13 | 1.13 |  | 1.23 |  | 1.37 |  | 1.52 |  | 1.69 |  | 1.90 |  |
| 14 | 1.15 |  | 1.22 |  | 1.29 |  | 1.38 |  | 1.46 |  | 1.52 |  |
| z-score | 0.334 | | 0.077 | | 0.026 | | 0.129 | | 0.180 | | 0.129 | |
