# Supplementary material for: The Beta Cell in Its Cluster: Stochastic Graphs of Beta Cell Connectivity in the Islets of Langerhans
Source: PLoS Comput Biol. 2015 Aug 12;11(8):e1004423. doi: 10.1371/journal.pcbi.1004423 (PMC4534467; doi:10.1371/journal.pcbi.1004423)
Supplement: S8 Table — (DOCX) [file pcbi.1004423.s034.docx]

|  | 8 | | 9 | | 10 | | 11 | | 12 | | 13 | |
| --- | --- | --- | --- | --- | --- | --- | --- | --- | --- | --- | --- | --- |
| Subj # | C | D | C | D | C | D | C | D | C | D | C | D |
| 1 | 1.28 | 1.16 | 1.57 | 1.37 | 1.98 | 1.67 | 2.54 | 2.11 | 3.22 | 2.71 | 3.81 | 3.45 |
| 2 | 1.15 | 1.25 | 1.34 | 1.54 | 1.58 | 2.01 | 1.88 | 2.71 | 2.24 | 3.63 | 2.65 | 5.20 |
| 3 | 1.23 | 1.30 | 1.49 | 1.55 | 1.84 | 1.89 | 2.28 | 2.34 | 2.80 | 2.84 | 3.40 | 3.48 |
| 4 | 1.14 | 1.05 | 1.32 | 1.09 | 1.54 | 1.15 | 1.82 | 1.22 | 2.13 | 1.29 | 2.45 | 1.35 |
| 5 | 1.27 | 1.08 | 1.70 | 1.16 | 2.42 | 1.24 | 3.51 | 1.32 | 5.13 | 1.38 | 7.23 | 1.45 |
| 6 | 1.09 | 1.09 | 1.18 | 1.19 | 1.29 | 1.35 | 1.44 | 1.51 | 1.61 | 1.73 | 1.80 | 1.95 |
| 7 | 1.13 | 1.27 | 1.33 | 1.61 | 1.63 | 2.14 | 2.03 | 3.01 | 2.51 | 4.25 | 3.09 | 5.72 |
| 8 | 1.11 | 1.20 | 1.31 | 1.38 | 1.60 | 1.60 | 2.05 | 1.87 | 2.69 | 2.17 | 3.63 | 2.50 |
| 9 | 1.20 | 1.14 | 1.45 | 1.29 | 1.80 | 1.50 | 2.30 | 1.75 | 2.89 | 2.02 | 3.62 | 2.29 |
| 10 | 1.25 | 1.38 | 1.51 | 1.79 | 1.86 | 2.36 | 2.27 | 3.19 | 2.71 | 4.03 | 3.25 | 4.95 |
| 11 | 1.21 | 1.28 | 1.43 | 1.55 | 1.75 | 1.89 | 2.17 | 2.31 | 2.69 | 2.90 | 3.28 | 3.52 |
| 12 | 1.15 | 1.12 | 1.31 | 1.27 | 1.51 | 1.47 | 1.74 | 1.76 | 2.04 | 2.15 | 2.36 | 2.58 |
| 13 | 1.12 |  | 1.22 |  | 1.35 |  | 1.51 |  | 1.68 |  | 1.90 |  |
| 14 | 1.13 |  | 1.21 |  | 1.28 |  | 1.36 |  | 1.43 |  | 1.51 |  |
| z-score | 0.283 | | 0.077 | | 0.129 | | 0.129 | | 0.180 | | 0.026 | |
