# Supplementary material for: The Beta Cell in Its Cluster: Stochastic Graphs of Beta Cell Connectivity in the Islets of Langerhans
Source: PLoS Comput Biol. 2015 Aug 12;11(8):e1004423. doi: 10.1371/journal.pcbi.1004423 (PMC4534467; doi:10.1371/journal.pcbi.1004423)
Supplement: S9 Table — (DOCX) [file pcbi.1004423.s035.docx]

|  | 8 | | 9 | | 10 | | 11 | | 12 | | 13 | |
| --- | --- | --- | --- | --- | --- | --- | --- | --- | --- | --- | --- | --- |
| Subj # | C | D | C | D | C | D | C | D | C | D | C | D |
| 1 | 1.36 | 1.18 | 1.67 | 1.39 | 2.04 | 1.63 | 2.37 | 1.92 | 2.69 | 2.24 | 2.99 | 2.55 |
| 2 | 1.19 | 1.22 | 1.41 | 1.42 | 1.61 | 1.70 | 1.87 | 2.00 | 2.10 | 2.38 | 2.37 | 2.74 |
| 3 | 1.29 | 1.30 | 1.53 | 1.53 | 1.79 | 1.77 | 2.05 | 2.01 | 2.27 | 2.26 | 2.46 | 2.44 |
| 4 | 1.19 | 1.10 | 1.36 | 1.14 | 1.55 | 1.20 | 1.71 | 1.28 | 1.91 | 1.35 | 2.10 | 1.40 |
| 5 | 1.35 | 1.14 | 1.79 | 1.25 | 2.43 | 1.32 | 3.25 | 1.41 | 3.93 | 1.51 | 4.49 | 1.59 |
| 6 | 1.14 | 1.14 | 1.22 | 1.26 | 1.34 | 1.39 | 1.47 | 1.52 | 1.62 | 1.64 | 1.75 | 1.79 |
| 7 | 1.18 | 1.27 | 1.39 | 1.55 | 1.64 | 1.92 | 1.98 | 2.30 | 2.31 | 2.64 | 2.61 | 2.92 |
| 8 | 1.15 | 1.24 | 1.34 | 1.42 | 1.58 | 1.65 | 1.86 | 1.90 | 2.15 | 2.08 | 2.44 | 2.31 |
| 9 | 1.23 | 1.17 | 1.47 | 1.33 | 1.76 | 1.49 | 2.06 | 1.67 | 2.38 | 1.83 | 2.73 | 1.97 |
| 10 | 1.30 | 1.47 | 1.55 | 1.94 | 1.89 | 2.45 | 2.20 | 3.06 | 2.48 | 3.56 | 2.77 | 3.91 |
| 11 | 1.27 | 1.39 | 1.50 | 1.71 | 1.78 | 2.11 | 2.05 | 2.54 | 2.33 | 2.97 | 2.58 | 3.35 |
| 12 | 1.16 | 1.15 | 1.32 | 1.31 | 1.42 | 1.49 | 1.56 | 1.67 | 1.70 | 1.87 | 1.88 | 2.12 |
| 13 | 1.16 |  | 1.27 |  | 1.43 |  | 1.56 |  | 1.71 |  | 1.87 |  |
| 14 | 1.19 |  | 1.27 |  | 1.34 |  | 1.45 |  | 1.54 |  | 1.57 |  |
| z-score | 0.283 | | 0.180 | | 0.129 | | 0.283 | | 0.334 | | 0.129 | |
