# Supplementary material for: The Beta Cell in Its Cluster: Stochastic Graphs of Beta Cell Connectivity in the Islets of Langerhans
Source: PLoS Comput Biol. 2015 Aug 12;11(8):e1004423. doi: 10.1371/journal.pcbi.1004423 (PMC4534467; doi:10.1371/journal.pcbi.1004423)
Supplement: S10 Table — * denotes p < 0.05 between the control and T2D groups, ** denotes a statistically-significant difference after the Bonferroni correction (using n = 64). (DOCX) [file pcbi.1004423.s036.docx]

|  | 8 | | 9 | | 10 | | 11 | | 12 | | 13 | |
| --- | --- | --- | --- | --- | --- | --- | --- | --- | --- | --- | --- | --- |
| Subj # | C | D | C | D | C | D | C | D | C | D | C | D |
| 1 | 1.94 | 0.78 | 2.38 | 1.20 | 2.37 | 1.37 | 2.27 | 1.38 | 2.06 | 1.32 | 1.90 | 1.26 |
| 2 | 1.46 | 1.17 | 2.26 | 1.60 | 2.64 | 1.70 | 2.76 | 1.65 | 2.69 | 1.53 | 2.51 | 1.35 |
| 3 | 1.94 | 1.38 | 2.64 | 1.70 | 2.88 | 1.80 | 2.82 | 1.74 | 2.61 | 1.67 | 2.40 | 1.54 |
| 4 | 0.93 | 0.27 | 1.41 | 0.39 | 1.66 | 0.54 | 1.75 | 0.68 | 1.71 | 0.75 | 1.65 | 0.81 |
| 5 | 8.23 | 0.30 | 11.04 | 0.47 | 10.47 | 0.56 | 8.52 | 0.65 | 6.57 | 0.66 | 5.04 | 0.68 |
| 6 | 0.65 | 0.63 | 1.02 | 1.03 | 1.31 | 1.38 | 1.55 | 1.56 | 1.66 | 1.66 | 1.68 | 1.66 |
| 7 | 1.08 | 3.09 | 1.75 | 3.96 | 2.11 | 3.90 | 2.16 | 3.40 | 2.08 | 2.77 | 1.91 | 2.29 |
| 8 | 1.19 | 1.46 | 2.09 | 1.92 | 2.64 | 2.20 | 2.64 | 2.27 | 2.39 | 2.23 | 2.11 | 2.14 |
| 9 | 1.83 | 0.63 | 2.60 | 0.95 | 2.82 | 1.13 | 2.79 | 1.21 | 2.65 | 1.21 | 2.37 | 1.20 |
| 10 | 1.85 | 2.59 | 2.43 | 3.01 | 2.64 | 2.93 | 2.56 | 2.52 | 2.39 | 2.26 | 2.21 | 2.03 |
| 11 | 2.74 | 1.68 | 3.75 | 2.05 | 4.17 | 2.15 | 4.13 | 2.10 | 3.89 | 1.95 | 3.49 | 1.81 |
| 12 | 1.75 | 0.93 | 2.67 | 1.52 | 3.16 | 1.86 | 3.29 | 2.02 | 3.35 | 2.01 | 3.18 | 1.92 |
| 13 | 1.01 |  | 1.49 |  | 1.85 |  | 2.12 |  | 2.23 |  | 2.25 |  |
| 14 | 0.56 |  | 0.72 |  | 0.88 |  | 0.95 |  | 1.02 |  | 1.06 |  |
| z-score | 1.414 | | 1.620 | | 1.826 | | 2.340* | | 2.495* | | 2.597** | |
