# Supplementary material for: The Beta Cell in Its Cluster: Stochastic Graphs of Beta Cell Connectivity in the Islets of Langerhans
Source: PLoS Comput Biol. 2015 Aug 12;11(8):e1004423. doi: 10.1371/journal.pcbi.1004423 (PMC4534467; doi:10.1371/journal.pcbi.1004423)
Supplement: S11 Table — (DOCX) [file pcbi.1004423.s037.docx]

|  | 8 | | 9 | | 10 | | 11 | | 12 | | 13 | |
| --- | --- | --- | --- | --- | --- | --- | --- | --- | --- | --- | --- | --- |
| Subj # | C | D | C | D | C | D | C | D | C | D | C | D |
| 1 | 4.78 | 2.28 | 5.92 | 3.53 | 5.95 | 4.01 | 5.68 | 3.94 | 4.97 | 3.57 | 4.46 | 3.22 |
| 2 | 2.67 | 4.42 | 4.18 | 6.11 | 4.89 | 6.14 | 5.08 | 5.52 | 4.94 | 4.76 | 4.59 | 3.83 |
| 3 | 4.70 | 3.85 | 6.47 | 4.71 | 7.10 | 4.93 | 6.87 | 4.74 | 6.24 | 4.49 | 5.65 | 3.99 |
| 4 | 1.98 | 0.41 | 3.14 | 0.63 | 3.66 | 0.91 | 3.91 | 1.16 | 3.78 | 1.24 | 3.62 | 1.37 |
| 5 | 13.09 | 0.38 | 17.59 | 0.62 | 16.65 | 0.77 | 13.49 | 0.90 | 10.30 | 0.93 | 7.78 | 0.96 |
| 6 | 1.34 | 1.34 | 2.20 | 2.26 | 2.85 | 3.19 | 3.43 | 3.50 | 3.65 | 3.72 | 3.63 | 3.71 |
| 7 | 2.58 | 6.94 | 4.34 | 8.91 | 5.38 | 8.66 | 5.47 | 7.42 | 5.17 | 5.82 | 4.63 | 4.58 |
| 8 | 3.65 | 2.86 | 6.58 | 3.82 | 8.36 | 4.38 | 8.25 | 4.49 | 7.20 | 4.42 | 6.07 | 4.17 |
| 9 | 3.97 | 1.68 | 5.76 | 2.54 | 6.17 | 3.02 | 6.07 | 3.25 | 5.66 | 3.22 | 4.92 | 3.15 |
| 10 | 3.61 | 5.84 | 4.83 | 6.95 | 5.27 | 6.75 | 5.09 | 5.83 | 4.73 | 5.13 | 4.27 | 4.42 |
| 11 | 4.88 | 3.95 | 6.73 | 4.97 | 7.47 | 5.26 | 7.39 | 5.20 | 6.90 | 4.73 | 6.14 | 4.27 |
| 12 | 2.78 | 2.17 | 4.26 | 3.58 | 5.07 | 4.50 | 5.24 | 4.85 | 5.33 | 4.76 | 5.02 | 4.43 |
| 13 | 1.99 |  | 3.02 |  | 3.74 |  | 4.35 |  | 4.58 |  | 4.63 |  |
| 14 | 1.07 |  | 1.42 |  | 1.75 |  | 1.85 |  | 2.04 |  | 2.17 |  |
| z-score | 0.540 | | 0.849 | | 1.260 | | 1.569 | | 2.186* | | 2.752** | |
