# Supplementary material for: The Beta Cell in Its Cluster: Stochastic Graphs of Beta Cell Connectivity in the Islets of Langerhans
Source: PLoS Comput Biol. 2015 Aug 12;11(8):e1004423. doi: 10.1371/journal.pcbi.1004423 (PMC4534467; doi:10.1371/journal.pcbi.1004423)
Supplement: S12 Table — (DOCX) [file pcbi.1004423.s038.docx]

|  | 8 | | 9 | | 10 | | 11 | | 12 | | 13 | |
| --- | --- | --- | --- | --- | --- | --- | --- | --- | --- | --- | --- | --- |
| Subj # | C | D | C | D | C | D | C | D | C | D | C | D |
| 1 | 0.741 | 0.467 | 0.886 | 0.706 | 0.854 | 0.811 | 0.832 | 0.836 | 0.832 | 0.852 | 0.822 | 0.851 |
| 2 | 0.455 | 0.582 | 0.649 | 0.793 | 0.746 | 0.905 | 0.821 | 0.951 | 0.799 | 0.951 | 0.771 | 0.910 |
| 3 | 0.563 | 0.664 | 0.727 | 0.830 | 0.768 | 0.896 | 0.796 | 0.882 | 0.789 | 0.862 | 0.777 | 0.843 |
| 4 | 0.428 | 0.167 | 0.580 | 0.225 | 0.696 | 0.290 | 0.707 | 0.355 | 0.707 | 0.406 | 0.707 | 0.428 |
| 5 | 0.932 | 0.254 | 1.178 | 0.369 | 1.151 | 0.425 | 1.027 | 0.490 | 0.945 | 0.487 | 0.904 | 0.513 |
| 6 | 0.292 | 0.304 | 0.407 | 0.464 | 0.509 | 0.552 | 0.573 | 0.664 | 0.633 | 0.696 | 0.668 | 0.704 |
| 7 | 0.502 | 0.639 | 0.765 | 0.814 | 0.862 | 0.869 | 0.891 | 0.849 | 0.898 | 0.828 | 0.875 | 0.828 |
| 8 | 0.414 | 0.586 | 0.690 | 0.713 | 0.852 | 0.832 | 0.881 | 0.873 | 0.881 | 0.857 | 0.867 | 0.857 |
| 9 | 0.635 | 0.323 | 0.837 | 0.493 | 0.944 | 0.583 | 0.964 | 0.623 | 0.968 | 0.632 | 0.942 | 0.640 |
| 10 | 0.712 | 1.163 | 0.877 | 1.280 | 0.953 | 1.251 | 0.927 | 1.071 | 0.887 | 1.000 | 0.882 | 0.975 |
| 11 | 0.597 | 0.994 | 0.770 | 1.159 | 0.850 | 1.205 | 0.863 | 1.151 | 0.858 | 1.103 | 0.836 | 1.061 |
| 12 | 0.366 | 0.413 | 0.558 | 0.666 | 0.622 | 0.771 | 0.680 | 0.841 | 0.692 | 0.865 | 0.733 | 0.874 |
| 13 | 0.387 |  | 0.519 |  | 0.648 |  | 0.692 |  | 0.725 |  | 0.736 |  |
| 14 | 0.286 |  | 0.338 |  | 0.406 |  | 0.466 |  | 0.466 |  | 0.466 |  |
| z-score | 0.026 | | 0.077 | | 0.129 | | 0.180 | | 0.129 | | 0.283 | |
