# Supplementary material for: The Beta Cell in Its Cluster: Stochastic Graphs of Beta Cell Connectivity in the Islets of Langerhans
Source: PLoS Comput Biol. 2015 Aug 12;11(8):e1004423. doi: 10.1371/journal.pcbi.1004423 (PMC4534467; doi:10.1371/journal.pcbi.1004423)
Supplement: S13 Table — (DOCX) [file pcbi.1004423.s039.docx]

|  | 8 | | 9 | | 10 | | 11 | | 12 | | 13 | |
| --- | --- | --- | --- | --- | --- | --- | --- | --- | --- | --- | --- | --- |
| Subj # | C | D | C | D | C | D | C | D | C | D | C | D |
| 1 | 2.50 | 2.39 | 2.98 | 2.74 | 3.68 | 3.18 | 4.35 | 3.77 | 5.15 | 4.40 | 5.81 | 4.97 |
| 2 | 2.29 | 2.51 | 2.59 | 2.89 | 2.93 | 3.51 | 3.34 | 4.22 | 3.82 | 4.99 | 4.40 | 5.99 |
| 3 | 2.44 | 2.46 | 2.81 | 2.82 | 3.27 | 3.23 | 3.81 | 3.74 | 4.46 | 4.22 | 5.11 | 4.78 |
| 4 | 2.31 | 2.10 | 2.60 | 2.19 | 2.94 | 2.28 | 3.31 | 2.37 | 3.77 | 2.52 | 4.17 | 2.60 |
| 5 | 2.58 | 2.23 | 3.27 | 2.37 | 4.38 | 2.48 | 6.07 | 2.57 | 8.38 | 2.75 | 11.3 | 2.88 |
| 6 | 2.20 | 2.22 | 2.34 | 2.35 | 2.54 | 2.56 | 2.74 | 2.77 | 2.97 | 3.04 | 3.28 | 3.35 |
| 7 | 2.27 | 2.50 | 2.57 | 3.06 | 2.97 | 3.93 | 3.51 | 5.18 | 4.05 | 6.85 | 4.70 | 8.63 |
| 8 | 2.22 | 2.32 | 2.55 | 2.63 | 2.91 | 2.93 | 3.58 | 3.29 | 4.45 | 3.67 | 5.47 | 4.10 |
| 9 | 2.38 | 2.28 | 2.77 | 2.50 | 3.30 | 2.80 | 3.92 | 3.12 | 4.54 | 3.45 | 5.38 | 3.74 |
| 10 | 2.43 | 2.66 | 2.80 | 3.25 | 3.24 | 3.98 | 3.77 | 5.06 | 4.32 | 5.93 | 4.93 | 6.80 |
| 11 | 2.41 | 2.64 | 2.75 | 3.08 | 3.22 | 3.58 | 3.80 | 4.13 | 4.47 | 4.80 | 5.25 | 5.41 |
| 12 | 2.28 | 2.22 | 2.54 | 2.44 | 2.83 | 2.72 | 3.21 | 3.07 | 3.60 | 3.54 | 4.08 | 4.04 |
| 13 | 2.23 |  | 2.41 |  | 2.61 |  | 2.79 |  | 3.04 |  | 3.34 |  |
| 14 | 2.24 |  | 2.38 |  | 2.41 |  | 2.58 |  | 2.68 |  | 2.76 |  |
| z-score | 0.386 | | 0.129 | | 0.129 | | 0.231 | | 0.283 | | 0.231 | |
