# Supplementary material for: The Beta Cell in Its Cluster: Stochastic Graphs of Beta Cell Connectivity in the Islets of Langerhans
Source: PLoS Comput Biol. 2015 Aug 12;11(8):e1004423. doi: 10.1371/journal.pcbi.1004423 (PMC4534467; doi:10.1371/journal.pcbi.1004423)
Supplement: S14 Table — (DOCX) [file pcbi.1004423.s040.docx]

|  | 8 | | 9 | | 10 | | 11 | | 12 | | 13 | |
| --- | --- | --- | --- | --- | --- | --- | --- | --- | --- | --- | --- | --- |
| Subj # | C | D | C | D | C | D | C | D | C | D | C | D |
| 1 | 2.48 | 2.46 | 2.97 | 2.86 | 3.69 | 3.43 | 4.45 | 4.23 | 5.48 | 5.28 | 6.35 | 6.34 |
| 2 | 2.28 | 2.63 | 2.57 | 3.06 | 2.93 | 3.93 | 3.37 | 5.09 | 3.89 | 6.45 | 4.51 | 8.53 |
| 3 | 2.42 | 2.48 | 2.81 | 2.87 | 3.29 | 3.37 | 3.90 | 3.99 | 4.65 | 4.58 | 5.43 | 5.43 |
| 4 | 2.33 | 2.05 | 2.60 | 2.17 | 3.00 | 2.29 | 3.40 | 2.38 | 3.93 | 2.58 | 4.41 | 2.66 |
| 5 | 2.57 | 2.23 | 3.26 | 2.36 | 4.39 | 2.54 | 6.10 | 2.64 | 8.52 | 2.78 | 11.65 | 2.94 |
| 6 | 2.18 | 2.24 | 2.32 | 2.38 | 2.54 | 2.59 | 2.75 | 2.88 | 3.02 | 3.20 | 3.38 | 3.57 |
| 7 | 2.27 | 2.50 | 2.59 | 3.07 | 3.01 | 4.00 | 3.60 | 5.39 | 4.26 | 7.41 | 5.10 | 9.78 |
| 8 | 2.22 | 2.32 | 2.58 | 2.62 | 2.98 | 2.94 | 3.75 | 3.34 | 4.86 | 3.76 | 6.28 | 4.26 |
| 9 | 2.39 | 2.28 | 2.78 | 2.53 | 3.38 | 2.91 | 4.06 | 3.28 | 4.80 | 3.71 | 5.84 | 4.10 |
| 10 | 2.45 | 2.68 | 2.81 | 3.27 | 3.27 | 4.06 | 3.84 | 5.22 | 4.44 | 6.25 | 5.18 | 7.47 |
| 11 | 2.40 | 2.56 | 2.74 | 2.99 | 3.23 | 3.49 | 3.84 | 4.05 | 4.55 | 4.87 | 5.41 | 5.68 |
| 12 | 2.29 | 2.22 | 2.55 | 2.45 | 2.85 | 2.73 | 3.26 | 3.15 | 3.66 | 3.72 | 4.20 | 4.35 |
| 13 | 2.22 |  | 2.39 |  | 2.60 |  | 2.78 |  | 3.03 |  | 3.36 |  |
| 14 | 2.23 |  | 2.37 |  | 2.40 |  | 2.63 |  | 2.68 |  | 2.76 |  |
| z-score | 0.694 | | 0.489 | | 0.231 | | 0.180 | | 0.180 | | 0.026 | |
