# Supplementary material for: The Beta Cell in Its Cluster: Stochastic Graphs of Beta Cell Connectivity in the Islets of Langerhans
Source: PLoS Comput Biol. 2015 Aug 12;11(8):e1004423. doi: 10.1371/journal.pcbi.1004423 (PMC4534467; doi:10.1371/journal.pcbi.1004423)
Supplement: S15 Table — (DOCX) [file pcbi.1004423.s041.docx]

|  | 8 | | 9 | | 10 | | 11 | | 12 | | 13 | |
| --- | --- | --- | --- | --- | --- | --- | --- | --- | --- | --- | --- | --- |
| Subj # | C | D | C | D | C | D | C | D | C | D | C | D |
| 1 | 2.56 | 2.33 | 2.99 | 2.60 | 3.63 | 2.93 | 4.06 | 3.32 | 4.32 | 3.62 | 4.57 | 3.88 |
| 2 | 2.33 | 2.36 | 2.70 | 2.66 | 2.94 | 3.01 | 3.16 | 3.32 | 3.49 | 3.69 | 3.85 | 4.08 |
| 3 | 2.52 | 2.43 | 2.81 | 2.73 | 3.19 | 3.02 | 3.45 | 3.36 | 3.71 | 3.68 | 3.91 | 3.91 |
| 4 | 2.28 | 2.17 | 2.59 | 2.23 | 2.78 | 2.25 | 3.05 | 2.35 | 3.35 | 2.39 | 3.58 | 2.46 |
| 5 | 2.79 | 2.22 | 3.42 | 2.37 | 4.30 | 2.42 | 5.35 | 2.48 | 6.09 | 2.72 | 6.55 | 2.80 |
| 6 | 2.25 | 2.16 | 2.38 | 2.29 | 2.53 | 2.46 | 2.71 | 2.49 | 2.85 | 2.62 | 2.97 | 2.82 |
| 7 | 2.28 | 2.49 | 2.52 | 2.97 | 2.87 | 3.49 | 3.29 | 4.00 | 3.60 | 4.38 | 3.90 | 4.58 |
| 8 | 2.21 | 2.32 | 2.45 | 2.64 | 2.70 | 2.87 | 3.08 | 3.14 | 3.40 | 3.39 | 3.69 | 3.62 |
| 9 | 2.34 | 2.28 | 2.72 | 2.44 | 3.04 | 2.64 | 3.39 | 2.87 | 3.68 | 3.07 | 4.01 | 3.22 |
| 10 | 2.39 | 2.61 | 2.76 | 3.21 | 3.15 | 3.76 | 3.55 | 4.67 | 3.92 | 5.20 | 4.14 | 5.45 |
| 11 | 2.49 | 2.74 | 2.80 | 3.20 | 3.15 | 3.69 | 3.47 | 4.25 | 3.77 | 4.70 | 4.04 | 5.07 |
| 12 | 2.19 | 2.23 | 2.38 | 2.41 | 2.53 | 2.68 | 2.69 | 2.88 | 2.90 | 3.13 | 3.04 | 3.39 |
| 13 | 2.25 |  | 2.47 |  | 2.67 |  | 2.87 |  | 3.05 |  | 3.27 |  |
| 14 | 2.26 |  | 2.40 |  | 2.41 |  | 2.48 |  | 2.68 |  | 2.74 |  |
| z-score | 0.180 | | 0.489 | | 0.334 | | 0.386 | | 0.231 | | 0.231 | |
