# Supplementary material for: The Beta Cell in Its Cluster: Stochastic Graphs of Beta Cell Connectivity in the Islets of Langerhans
Source: PLoS Comput Biol. 2015 Aug 12;11(8):e1004423. doi: 10.1371/journal.pcbi.1004423 (PMC4534467; doi:10.1371/journal.pcbi.1004423)
Supplement: S16 Table — (DOCX) [file pcbi.1004423.s042.docx]

|  | **Degree** | | | | | | **Number of Cells per Component** | | | | | |
| --- | --- | --- | --- | --- | --- | --- | --- | --- | --- | --- | --- | --- |
|  | **Control** | | | **T2D** | | | **Control** | | | **T2D** | | |
| **Radius** | **Large** | **Small** | **Diff** | **Large** | **Small** | **Diff** | **Large** | **Small** | **Diff** | **Large** | **Small** | **Diff** |
| 8 | 0.32 | 0.36 | -0.04 | 0.36 | 0.39 | -0.03 | 1.19 | 1.22 | -0.03 | 1.22 | 1.24 | -0.02 |
| 9 | 0.59 | 0.6 | -0.01 | 0.62 | 0.64 | -0.02 | 1.41 | 1.42 | -0.01 | 1.44 | 1.46 | -0.02 |
| 10 | 0.86 | 0.83 | 0.03 | 0.9 | 0.86 | 0.04 | 1.71 | 1.66 | 0.05 | 1.75 | 1.7 | 0.05 |
| 11 | 1.13 | 1.02 | 0.11 | 1.16 | 1.05 | 0.11 | 2.09 | 1.9 | 0.19 | 2.16 | 1.96 | 0.2 |
| 12 | 1.36 | 1.19 | 0.17 | 1.41 | 1.22 | 0.19 | 2.53 | 2.14 | 0.39 | 2.65 | 2.21 | 0.44 |
| 13 | 1.58 | 1.33 | 0.25 | 1.64 | 1.37 | 0.27 | 3.05 | 2.37 | 0.68 | 3.18 | 2.45 | 0.73 |
