# Supplementary material for: The Beta Cell in Its Cluster: Stochastic Graphs of Beta Cell Connectivity in the Islets of Langerhans
Source: PLoS Comput Biol. 2015 Aug 12;11(8):e1004423. doi: 10.1371/journal.pcbi.1004423 (PMC4534467; doi:10.1371/journal.pcbi.1004423)
Supplement: S17 Table — For each islet type, the measures were calculated for the random simulation (Sim) and compared to the experimental (Exp) measure results. The difference (Diff) between the two values is given. (DOCX) [file pcbi.1004423.s043.docx]

|  | Mean Degree | | | Mean # Components | | | Mean # NS Components | | | Mean # Cells/Component | | | Mean # Cells/NS Component | | |
| --- | --- | --- | --- | --- | --- | --- | --- | --- | --- | --- | --- | --- | --- | --- | --- |
|  | Sim | Exp | Diff | Sim | Exp | Diff | Sim | Exp | Diff | Sim | Exp | Diff | Sim | Exp | Diff |
| C,large | 1.65 | 0.88 | 0.76 | 10.37 | 22.62 | -12.25 | 4.70 | 7.34 | -2.65 | 3.79 | 1.74 | 2.05 | 7.16 | 3.27 | 3.89 |
| T2D,large | 1.69 | 0.93 | 0.76 | 8.11 | 17.96 | -9.85 | 3.74 | 5.93 | -2.20 | 4.00 | 1.81 | 2.20 | 7.52 | 3.44 | 4.07 |
| C,small | 1.02 | 0.89 | 0.14 | 2.51 | 2.78 | -0.28 | 1.00 | 1.03 | -0.03 | 1.93 | 1.74 | 0.19 | 3.32 | 2.98 | 0.34 |
| T2D,small | 1.05 | 0.92 | 0.13 | 2.56 | 2.83 | -0.27 | 1.03 | 1.08 | -0.05 | 1.97 | 1.79 | 0.19 | 3.43 | 3.06 | 0.37 |
