# Supplementary figures and images for: The Beta Cell in Its Cluster: Stochastic Graphs of Beta Cell Connectivity in the Islets of Langerhans
Source: PLoS Comput Biol. 2015 Aug 12;11(8):e1004423. doi: 10.1371/journal.pcbi.1004423 (PMC4534467; doi:10.1371/journal.pcbi.1004423)

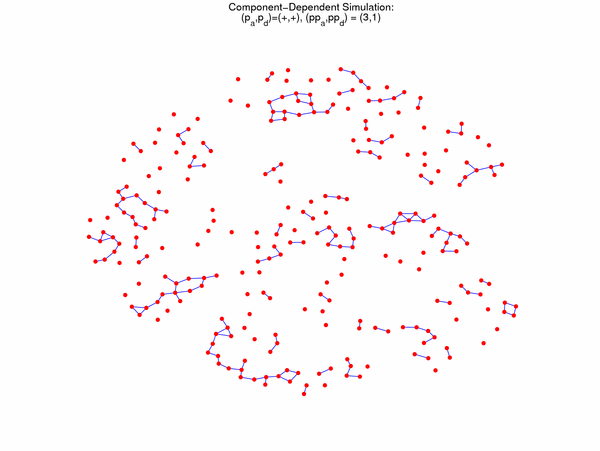

Supplement: S1 Video — (GIF) [file pcbi.1004423.s044.gif]

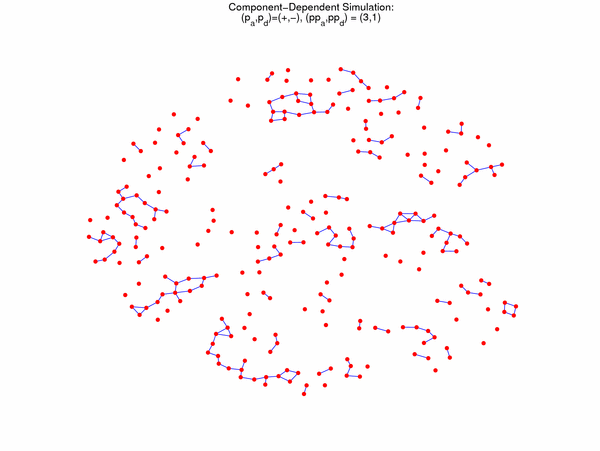

Supplement: S2 Video — (GIF) [file pcbi.1004423.s045.gif]

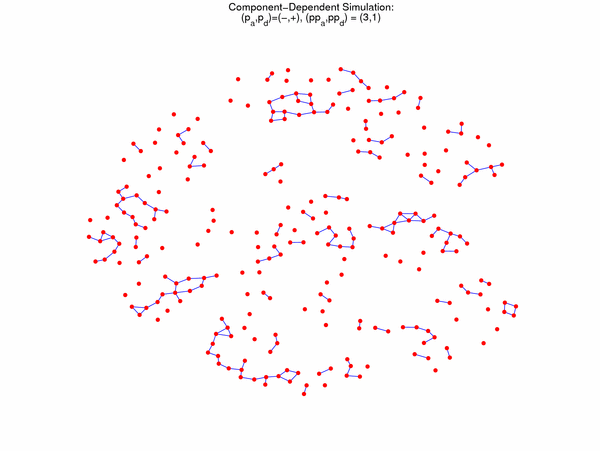

Supplement: S3 Video — (GIF) [file pcbi.1004423.s046.gif]

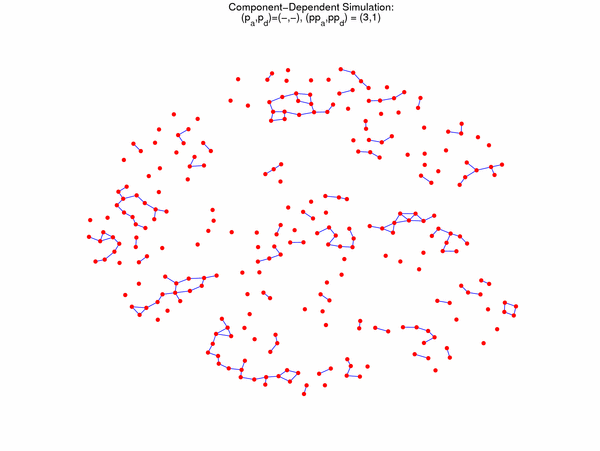

Supplement: S4 Video — (GIF) [file pcbi.1004423.s047.gif]
